# Identification of highly potent and selective inhibitor, TIPTP, of the p22phox-Rubicon axis as a therapeutic agent for rheumatoid arthritis

**DOI:** 10.1038/s41598-020-61630-x

**Published:** 2020-03-12

**Authors:** Ye-Ram Kim, Jae-Sung Kim, Su-Jin Gu, Sungsin Jo, Sojin Kim, Sun Young Kim, Daeun Lee, Kiseok Jang, Hyunah Choo, Tae-Hwan Kim, Jae U. Jung, Sun-Joon Min, Chul-Su Yang

**Affiliations:** 1 Department of Molecular & Life Science, Hanyang University, Ansan, 15588 South Korea; 2 Department of Bionano Technology, Hanyang University, Seoul, 04673 South Korea; 3 Department of Chemical & Molecular Engineering/Applied Chemistry, Ansan, 15588 South Korea; 4 Hanyang University Hospital for Rheumatic Diseases, Seoul, 04763 South Korea; 5 Department of Pathology, Hanyang University College of Medicine, Seoul, 04763 South Korea; 6 Center for Neuro-Medicine, Brain Science Institute, Korea Institute of Science and Technology (KIST), Seoul, 02792 South Korea; 7 Department of Molecular Microbiology and Immunology, Keck School of Medicine, University of Southern California, Los Angeles, CA 90089 USA

**Keywords:** Autoimmunity, Experimental models of disease

## Abstract

Rheumatoid arthritis (RA) is a chronic inflammatory autoimmune disease linked to oxidative stress, which is associated with significant morbidity. The NADPH oxidase complex (NOX) produces reactive oxygen species (ROS) that are among the key markers for determining RA’s pathophysiology. Therefore, understanding ROS-regulated molecular pathways and their interaction is necessary for developing novel therapeutic approaches for RA. Here, by combining mouse genetics and biochemistry with clinical tissue analysis, we reveal that *in vivo* Rubicon interacts with the p22phox subunit of NOX, which is necessary for increased ROS-mediated RA pathogenesis. Furthermore, we developed a series of new aryl propanamide derivatives consisting of tetrahydroindazole and thiadiazole as p22phox inhibitors and selected 2-(tetrahydroindazolyl)phenoxy-*N-(*thiadiazolyl)propanamide **2** (TIPTP, M.W. 437.44), which showed considerably improved potency, reaching an IC_50_ value up to 100-fold lower than an inhibitor that we previously synthesized reported N8 peptide-mimetic small molecule (blocking p22phox–Rubicon interaction). Notably, TIPTP treatment showed significant therapeutic effects a mouse model for RA. Furthermore, TIPTP had anti-inflammatory effects *ex vivo* in monocytes from healthy individuals and synovial fluid cells from RA patients. These findings may have clinical applications for the development of TIPTP as a small molecule inhibitor of the p22phox-Rubicon axis for the treatment of ROS-driven diseases such as RA.

## Introduction

Rheumatoid arthritis (RA) presents with characteristic chronic synovial hyperplasia and inflammation, and is a type of systemic autoimmune disease. Besides, in RA is also associated with invasion of inflammation and hyperplasia into the adjacent bone and cartilage, which cause slow degeneration of the knee joints^1^. The current drugs for treating RA, such as disease-modifying anti-rheumatic drugs (methotrexate), nonsteroidal anti-inflammatory drugs (NSAID), steroids (prednisone), glucocorticoids, immunosuppressants, and biological therapies (TNF-α and IL-1 activity blocking monoclonal antibodies), have dramatically improved prognosis^2,3^. Nevertheless, about 20–40% proportion of patients fail to respond to current therapies, and biological therapies raises the risk of serious infection^1,4,5^. Therefore, attention has urgently focused on alternative RA therapeutics with high efficacy and reduced side effects.

Arthritis is an umbrella term used to describe inflammation of the joints. However, there are different kinds of arthritis, including RA and osteoarthritis (OA). Although RA and OA both affect the joints, they are very different forms of the same broader condition. Accumulating evidence has shown that reactive oxygen species (ROS) is considered to play a role in the pathophysiology of RA, but not OA^6–14^. Thus, in RA patients the antioxidant system is deranged leading to significantly elevated production of ROS, which in turn causes oxidative damage to DNA and proteins and lipid peroxidation and these culminate in the pathogenesis of chronic tissue degeneration^6–8,15^. The elevated ROS contribute to the production of rheumatoid factor by oxidizing IgG, and also cause hyaluronic acid depolymerization, thereby reducing the viscosity of the joints. In addition, ROS also render T cells to be hypo-responsiveness by affecting protein stability and enhancing proteasomal degradation^9^. Furthermore, enhanced ROS production may damage cartilage and cause bone resorption. Several studies in RA patients showed accelerated lipid peroxidation in the synovial fluid, serum, and erythrocytes^6,10,11^. and in their synovial fluid and tissues, oxidation of low-density lipoproteins, elevated levels of lipid peroxidation products, and the presence of protein carbonyl groups have been observed^12–14^.

ROS form in the inflamed joints of RA patients via chondrocytes, activated macrophages in the synovial membrane, and activated neutrophils in the synovial cavity^16^. Superoxide anion (O_2_^−^) formed by NADPH oxidase (NOX) is a predominant ROS that contributes to inflammation in RA patients^17^. NOX is a complex membrane protein that is made up of one subunit, p22phox, which is an integral membrane protein, gp91phox, the catalytic subunit, and the regulatory subunits p40phox, p47phox, p67phox, along with the small GTPase Rac^18,19^. Importantly, ROS mediate their effects by activating the signaling pathways of NF-kB and NLRP3 inflammasome. These signaling cascades control the synthesis and release of inflammatory cytokines and ultimately lead to inflammation of joints and also the destruction of macrophages in animals and patients with RA^6,20–22^. Collectively these findings suggest that placing the production of ROS under control is a potentially viable therapeutic approach to treat RA. Although ROS have been found to be necessary for RA’s pathophysiology, the molecular targets have yet to be definitively characterized. Thus, there is still an urgent need for the development of a NOX-regulated novel therapeutic target for treating RA.

We previously discovered that Run/cysteine-rich-domain-containing Beclin1-interacting autophagy protein (Rubicon) is an essential positive regulator of the NOX complex; it interacts with p22phox of NOX upon microbial infection, and facilitates the stabilization and phagosomal trafficking of the p22phox–NOX complex to induce a ROS burst, inflammatory cytokine production, and thereby potent anti-microbial activities^18^. Furthermore, we recently reported an N-terminal eight-amino-acid N8 peptide derived from p22phox and a mimetic compound from *in silico* virtual screening that interferes with the interaction between Rubicon and p22phox, to strongly suppress the production of ROS and inflammatory cytokines. These effects helped to considerably curtail the mortality in mice suffering with polymicrobial sepsis induced by cecal ligation procedure (CLP)^23^. In this regard, the previously^23^ reported the N8 peptidomimetic we described before, which has strong anti-inflammatory and antioxidative effects, proves to be an important resource for the development of a therapeutic against RA.

In this study, we identified that *in vivo* p22phox interacts with Rubicon, which is necessary for increased ROS-mediated murine RA pathogenesis. Furthermore, we developed a TIPTP (p22 inhibitor) that showed considerably improved potency and selectivity than the previously reported N8 peptide-mimetic small molecule [23 Particularly, we show that NLRP3 inflammasomes induced by ROS, *in vivo*, can be inhibited by targeting the p22hox–Rubicon interaction by p22 inhibitor, which also showed enhance therapeutic effects *ex vivo* on monocytes from healthy individuals and synovial fluid cells from RA patients, and in mouse models for RA. Thus, the selective inhibition of p22hox–Rubicon, which may be desirable from a safety perspective, is not only achievable pharmacologically, but also efficacious at inhibiting inflammatory diseases in preclinical models.

## Materials and Methods

### Materials

LPS (*Escherichia coli* O111:B4) and ATP were purchased from Sigma. Specific antibodies against Rubicon (ab92388) were purchased from Abcam. Antibodies against Beclin-1 (3738) and UVRAG (5320) were purchased from Cell Signaling Technology. Abs specific for gp91-phox (54.1), p22-phox (CS9), p47-phox (A-7), p67-phox (H-300), p40-phox (D-8), NOX1 (C-10), TLR4 (25), TRAF6 (D-10), IL-1β (B122), IL-18 (H-173), Caspase-1 (M-20), ASC (B-3), V5 (H-9), Flag (D-8) and actin (I-19) were purchased from Santa Cruz Biotechnology. NLRP3 (Cryo-2) were purchased from AdipoGen. NOX3 (bs-3683R) were purchased from Bioss Inc. NOX4 (NB110–58849) and NOX5 (NBP1–68862) were purchased from Novus Biologicals.

### Cells

The mouse macrophage cell line RAW264.7 (ATCC TIB-71; American Type Culture Collection) and HEK293T (ATCC-11268) cells were maintained in DMEM (Invitrogen) containing 10% FBS (Invitrogen), sodium pyruvate, nonessential amino acids, penicillin G (100 IU/ml), and streptomycin (100 μg/ml). Transient transfections were performed with Lipofectamine 3000 (Invitrogen), or calcium phosphate (Clontech), according to the manufacturer’s instructions. Raw264.7 stable cell lines were generated using a standard selection protocol with 2 μg/ml of puromycin. Mouse primary bone marrow derived-macrophages (BMDMs) were isolated from C57BL/6 mice and cultured in DMEM for 3–5 days in the presence of 25 ng/ml recombinant macrophage colony stimulating factor (R&D Systems, 416-ML, Minneapolis, MN, USA), as described previously^23^. Human adherent monocytes were prepared from PBMCs donated by healthy subjects, as described previously^19^.

For synovial fluid containing synoviocytes were collected according to a previously described protocol^24–26^. Briefly, after excision of the skin and patellar ligament under a dissecting microscope to expose the synovial membrane, a 30-gauge needle (BD Biosciences, San Jose, CA, USA) was carefully inserted into the membrane, and the synovial cavity was washed by repetitive injections and aspirations with PBS (20 μl) to obtain synovial lavage material. This procedure was repeated five times, and a total volume of 100 μl of synovial lavage fluid was obtained. After that step, joint and paws samples were removed and kept in RPMI 1640 medium containing 10% FBS, 100 IU/ml penicillin, 100 μg/ml streptomycin, and 1 mg/ml collagenase (Sigma-Aldrich). The entire mixture was minced and incubated for 1 hour at 37 °C in a 5% CO_2_ atmosphere. The procedure was repeated three times, and cell suspensions were filtered with a cell strainer after red blood cell lysis. This method usually yields 3 ∼ 10 × 10^4^ cells from arthritic mice. Synovial fluid containing fibroblast-like and macrophage-like synoviocytes^27^.

Synovial tissue specimens were obtained from all female patients with RA (n = 16, 60.5 years ± 6.0) or OA (n = 10, 59.5 years ± 7.2) during open synovectomy or joint replacement surgery at Hanyang University Hospital. All patients gave informed consent, and the procedure was approved by the Ethics Committee of Hanyang University Hospital approved this study (2017-05-003). This study was approved by the bioethics committee of The Red Cross (Seoul, South Korea), which oversees studies using samples from human subjects, and all methods were performed in accordance with the relevant guidelines and regulations.

### Mouse model of rheumatoid arthritis

The collagen-induced arthritis mouse model was established as previously described^28^. Briefly, male DBA/1 J mice (6 − 8-weeks-old) were obtained from Beijing Vital River Laboratory Animal Technology Co., Ltd. (Charles River Laboratories department in Beijing, China), and kept under specific pathogen-free conditions. Chick collagen type II (Chondrex, Redmond, Washington, USA) was dissolved in 0.05 M acetic acid to a concentration of 2 mg/mL and emulsified with a complete Freund’s adjuvant (2 mg/mL *M. tuberculosis* H37Ra, Chondrex). At the beginning of the experiments (day 0), the mice were immunized with a 0.1 mL emulsion containing 100 μg of collagen at the tail base with a glass syringe and 25-G needles and then administered a booster (on day 21) with the same preparation of collagen and incomplete Freund’s adjuvant (Chondrex).

Collagen antibody-induced arthritis mouse model was induced as previously described^29^. Mice received a single‐dose intravenous injection of anti-type II collagen antibody cocktail (8 mg/mouse) (Arthrogen‐CIA Arthritogenic Monoclonal Antibody, no. 53010; Chondrex) on day −3 and an intraperitoneal injection of 100 μg of lipopolysaccharide on day 0 and were then monitored for the indicated times. Mice were sacrificed under anesthesia for the indicated times and knee joints were isolated. All animal-related procedures were reviewed and approved by the Institutional Animal Care and Use Committee of the Hanyang University (protocol 2017–0219). All animal experiments were performed in accordance with Korean Food and Drug Administration guidelines. The animals were fed standard rodent food and water ad libitum, and housed (maximum of 5 per cage) in sawdust-lined cages in an air-conditioned environment with 12-hour light/dark cycles. Animal husbandry was provided by the staff of the IACUC under the guidance of supervisors who are certified Animal Technologists, and by the staff of the Animal Core Facility. Veterinary care was provided by IACUC faculty members and veterinary residents located on Hanyang University. Details of compound synthesis, microsomal and plasma stability assays, histology, immunohistochemistry, and histopathologic and histomorphogenic analyses, are provided in the Supplementary Information.

### Protein purification and mass spectrometry

To identify p22phox-binding proteins, precipitates were washed extensively with lysis buffer^23^. Proteins bound to beads were eluted and separated on a NuPAGE 4–12% Bis-Tris gradient gel (Life Technologies). After silver staining (Life Technologies), a gel section containing the pure protein was subjected to tryptic digestion and analyzed by ion-trap mass spectrometry at the Harvard Taplin Biological Mass Spectrometry facility, and amino acid sequences were determined by tandem mass spectrometry and database searches. Briefly, the extracted protein (10 μg) from the gel slice was added to 100 mM ammonium bicarbonate (pH 8). This was then incubated with dithiothreitol (10 mM) at 56 °C for 30 minutes. After cooling to room temperature, the cysteine residues were alkylated using iodoacetamide (50 mM). Trypsin gold (Promega, UK) was subsequently added and the samples were incubated overnight at 37 °C. The digested peptides were concentrated and separated using an Ultimate 3000 HPLC series (Dionex, USA). Samples were then trapped on an Acclaim PepMap 100 C18 LC column, 5um, 100 A 300um i.d. ×5 mm (Dionex, USA), then further separated in Nano Series Standard Columns 75μm i.d. ×15 cm. This was packed with C18 PepMap100 (Dionex, USA) and a gradient from 3.2%–44% (v/v) solvent B (0.1% formic acid in acetonitrile) over 30 minutes was used to separate the peptides. The digested peptides were eluted (300nL/min) using a triversa nanomate nanospray source (Advion Biosciences, USA) into a LTQ Orbitrap Elite Mass Spectrometer (ThermoFisher Scientific, Germany). The MS and MS/MS data were then searched against Uniprot using Sequest algorithm and the partial sequence was then compared to the other similar protein sequences available in the protein database.

### Immunofluorescence (IF) and Immunohistochemistry (IHC)

Synovial tissues were fixed in 10% formalin for about 2 weeks, and embedded in paraffin. Staining methods were previously described in detail^30^. Briefly, tissue slides (5 μm thick) were deparaffinized, dehydrated, incubated with proteinase K (Abcam, ab64220), permeablized with TBS-T (0.3% Triton X-100), and eliminated endogenous peroxidase with BLOXALL (Vector Lab, SP-6000). They were then incubated for overnight at 4 °C with the appropriate primary antibodies in antibody diluent (DAKO, S3022). For immunofluorescence, the incubated slide was visualized using secondary antibodies: alexa 488-conjugated anti-mouse antibody (Invitrogen, A11001) and Cy3-conjugated anti-rabbit antibody (Jackson Immunoresearch, 111-165-144). Nuclei were counterstained with DAPI (Thermo Scientific, p36935). To visualize stained cells, confocal microscope (Leica Microsystems, Wetzlar, Germany) was used. For immunohistochemistry, the incubated slide was followed by ABC kit components (Vector Lab, PK-6102), DAB substrate kit (Vector Lab, sk4100), counterstaining with hematoxylin (Merck, 1.05174.0500), and mounting with Permanent mounting medium (Vector Lab, H-5000). To visualize stained cells, images were collected with a Nikon eclipse Ti-U microscope.

### Immunoblot and immunoprecipitation analysis

Immunoblot and immunoprecipitation assays were performed as described previously^18,23^. For immunoprecipitation, cells were harvested and then lysed in NP-40 buffer supplemented with a complete protease inhibitor cocktail (Roche). After pre-clearing with protein A/G agarose beads (GE Healthcare Life Science) for 1 h at 4 °C, whole-cell lysates were used for immunoprecipitation with the indicated antibodies. Generally, 1–4 μg of commercial antibody was added to 1 ml of cell lysates and incubated at 4 °C for 8 to 12 h. After the addition of protein A/G agarose beads for 6 h, immunoprecipitants were extensively washed with lysis buffer and eluted with SDS loading buffer by boiling for 5 min.

For immunoblotting, polypeptides were resolved by SDS-polyacrylamide gel electrophoresis and transferred to a PVDF membrane (Bio-Rad). Immuno detection was achieved with specific antibodies. Antibody binding was visualized by chemiluminescence (ECL; Millipore) and detected by a Vilber chemiluminescence analyzer (Fusion SL 3; Vilber Lourmat).

### Enzyme-linked immunosorbent assay

Cell culture supernatants, mice sera and synovial fluid were analyzed for cytokine content using the BD OptEIA ELISA set (BD Pharmingen) for the detection of TNF-α, IL-6, IL-1β, and IL-18. All assays were performed as recommended by the manufacturer^23^.

### Flow cytometric measurement of ROS production

Intracellular ROS levels were measured by flow cytometry from cells cultured in serum-free medium and loaded with the redox-sensitive dye 2 µM dihydroethidium (DHE for O_2_^−^; Calbiochem) or 10 µM 2′,7′-Dichlorofluorescin diacetate (DCFH-DA for H_2_O_2_; Calbiochem)^18,19^. The cells were thoroughly and quickly washed with pulse spin and immediately acquired for analyses in FACSCalibur (BD Biosciences, San Jose, CA). The data were plotted using CellQuest software (BD Biosciences).

### Measurement of intracellular ROS by NOX activity

Intracellular superoxide production was measured by the lucigenin (bis-*N*-methylacridinium nitrate)-ECL method^18,19^. Briefly, cells lysates were allowed to equilibrate for 30 min at 37 °C in a reaction containing 50 mM phosphate buffer (pH 7.0), 1 mM EGTA, 150 mM sucrose, and a protease inhibitor mixture prior to the addition of Krebs-HEPES buffer containing lucigenin (5 μM) as the electron acceptor and NADPH (100 μM) as the electron donor. The values are expressed as relative light units per 1 × 10^5^ cells.

### Plasmid

The plasmid encoding full-length of the p22phox (V5-p22phox) and Rubicon (Flag-Rubicon) were previously described^23^.

### Construction of Adenoviral shRubicon or Rubicon

An adenovirus expressing short hairpin RNA (shRNA) to the Rubicon gene was constructed using the AdEasy system (Stategene)^18^. The shRNA oligonucleotides sequences were as follows: 5′-gatccc g cat att cgc tcc cac tcg g ttcaagaga g cca gca gct ccc agt tca g tttttggaaa-3′, 3′-gg c cga gtg gga gcg aat atg c aagttctct c tga act ggg agc tgc tgg c aaaaaaccttttcga-5′. These dsDNA oligonucleotides were cloned into the pSuper vector (OligoEngine) between the *BglII* and *HindIII* restriction sites containing the mouse U6 promoter. The double-strand shRNA oligonucleotides containing the termination signal were inserted at the 3′ end of the mouse U6 promoter and subcloned into the pShuttle vector (Stategene) *NotI* and *HindIII* restriction sites. Control vector was constructed by inserting a sequence that expresses an shRNA with limited homology to the Rubicon sequences. DNA fragments corresponding to the coding sequences of the Rubicon genes were amplified by PCR and subcloned into the pShuttle-CMV vector (Stategene) between the *NotI* and *EcoRV* restriction sites.

### Adenovirus production

Recombinant adenoviruses were constructed using AdEasy system (Stategene): digested adenovirus vectors with the *Pac I* were transfected into the AD-293 producer cells in a 6-well plate and cultured with fresh media until cytopathic effect (CPE) was observed^18^. When 80% CPE were observed, recombinant adenoviruses were harvested by repeatedly freezing at −80 °C and thawing at 37 °C for four times. Cell lysates were then centrifuged at 2,000 g for 30 min at 25 °C and the supernatants containing recombinant adenovirus particles were stored at −80 °C. All adenoviruses were propagated in AD-293 cells, purified and concentrated by BD Adeno-X^TM^ purification kit (BD Biosciences Clontech). The typical titers were in the range of 10^12^–10^13^ plaque-forming units (pfu)/mL as determined via plaque assay using 1.25% SeaPlaque GTG agarose (BioWhittaker Molecular Applications) overlay. A sterile carrier solution [phosphate-buffered saline] was used for control injections and dilution of the viruses. All adenovirus-related procedures were reviewed and approved by Institutional Biosafety Committee of the Hanyang University (HY-IBC-2017-01).

### Injection of recombinant adenoviruses for depletion or expression of Rubicon in mice

Recombinant adenoviruses (Ad-vector, Ad-shRubicon, and Ad-Rubicon) were injected intravenously via the tail vein at a dose of ~1 × 10^13^ pfu/mouse twice. At 2 days post-transduction, mice were challenged with collagen antibody-induced arthritis (CAIA). All animal-related procedures were reviewed and approved by the Institutional Animal Care and Use Committee of the Hanyang University (protocol 2017–0024).

### Synthesis of TIPTP

To investigate new chemical inhibitor of p22phox, we generally synthesized propanamide derivatives in five steps starting from tetrahydroindazole. The detail experimental procedure for the synthesis of **TIPTP** is provided in Supplementary Information. The detail experimental procedure for biochemical properties (plasma stability, microsomal stability, and CYP inhibition) and pharmacokinetic profile are provided in Supplementary Information.

### Statistical analysis

Data obtained from independent experiments (means ± SD) were analyzed using a two-tailed Student’s *t*-test with Bonferroni adjustment or ANOVA for multiple comparisons. Differences were considered significant at *p* < 0.05. For survival, data were graphed and analyzed by the product limit method of Kaplan and Meier, using the log-rank (Mantel–Cox) test for comparisons (Prism, version 5.0, GraphPad Software).

## Results

### p22phox interaction with Rubicon during RA progression

**Figure 1:**
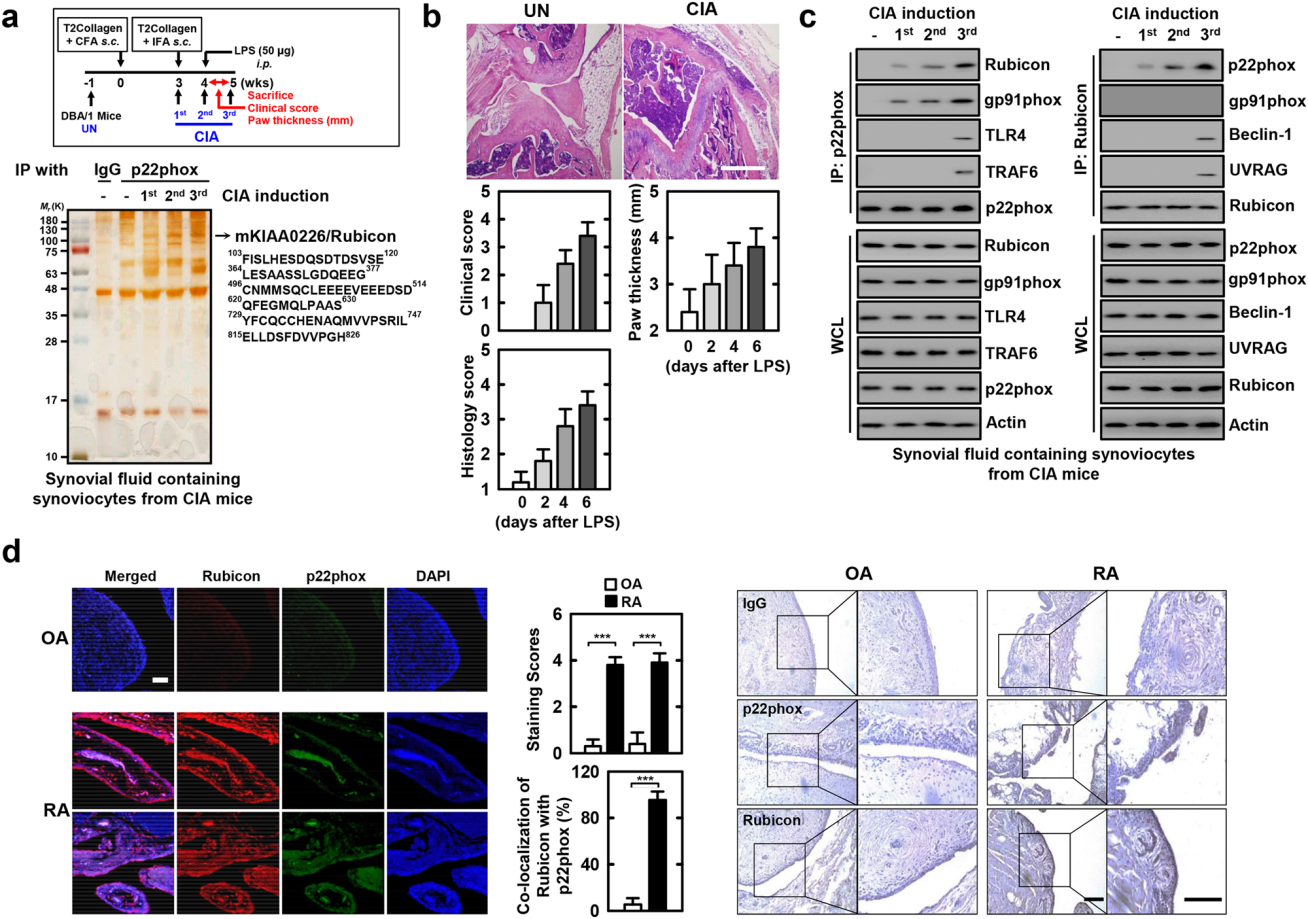
p22phox interaction with Rubicon in human and CIA mice. (**a**) Schematic of the collagen-induced RA (CIA) model (upper). p22phox complexes purified from synovial fluid containing synoviocytes from CIA mice were subjected to mass spectrometry analysis. Silver stained gel (bottom left) and peptides identified by mass spectrometry analysis (bottom right) The red-colored letters indicate the peptides identified from mass spectrometry analysis. (**b**) Representative Hematoxylin-Eosin (H&E) staining of the ankle joints of each group (upper); Scale bar, 500 μm. Clinical arthritis, swelling of paws scores (middle), and histopathology scores (bottom). Results are expressed as means ± SD (5 mice per group). (**c**) Synovial fluid containing synoviocytes from CIA mice for the indicated times, followed by IP with αp22phox (left) or αRubicon (right), followed by IB with αRubicon, αp22phox, and αgp91phox. The anti-p22phox blot was also tested for αTLR4, αTRAF6 binding. The anti-Rubicon blot was also tested for αBeclin-1, or αUVRAG. WCLs were used for IB with αRubicon, αp22phox, αgp91phox, αTLR4, αTRAF6, αBeclin-1, αUVRAG. Actin Western blot was used as a loading control. (**d**) OA and RA cells were stained with αp22phox (Alexa Fluor 488; green) and αRubicon (Cy3; red). Nuclei were counterstained with DAPI. Cells were visualized by confocal microscopy (left). The middle panel shows the quantitative data of staining intensity of p22phox and Rubicon (upper) and the colocalization index (%) between p22phox and Rubicon (bottom). Immunohistochemical analysis to examine p22phox-DAB (3,3′-diaminobenzidine) and Rubicon-AEC (3-amino-9-ethylcarbazole) expression (right). Representative images from six independent OA and RA patients are shown. Insets, enlargement of outlined areas. Scale bars: 200 μm. The data are representative of five independent experiments with similar results (**a**, **c** and **d**). Statistical analysis was done using the Student’s *t*-test with Bonferroni adjustment. Data are considered different at p < 0.05. ***p < 0.001. Experimental procedures were described in Supplementary Information.

Among the NOX subunits, p22phox plays a central organizing role, and its structure forms a highly regulated and interactive subunit as an anchor for NOX activation^19,31^. To establish a role for p22phox in intracellular signaling pathways as a therapeutic strategy for RA in synoviocytes, we investigated whether p22phox interacts with molecules involved in RA progression. The p22phox complexes were subjected to co-immunoprecipitation (co-IP) with synovial fluid containing synoviocytes from collagen-induced arthritis (CIA) mice (Fig. 1a, top). The purified p22phox complexes selectively retrieved a major endogenous protein with a molecular weight of 130 kDa. The protein was identified as Rubicon (130 K) by mass spectrometry followed by peptide sequencing (Fig. 1b).

Endogenous co-IP showed that p22phox interacted gradually and then strongly with endogenous Rubicon, but not with components of phagocytosis-mediated innate immunity (TLR4 and TRAF6). The anti-Rubicon co-IP bound with p22phox but not the autophagy complex (Beclin-1 and UVRAG) during RA progression in synovial fluid containing synoviocytes from CIA mice (Fig. 1c). Notably, p22phox interact with NOX2/gp91phox, and NOX4 on CIA induction-dependent manner (Fig. 1c and Supplemental Fig. 1). In CIA-induced RA, p22phox interacts with Rubicon and NOX2 in macrophage-like synoviocytes, because of, NOX2 expressed macrophage-like synoviocytes^27^. As previously reported^18,23^, p22phox associates with Rubicon in oxidative stress-mediated disease.

Arthritis is an umbrella term used to describe inflammation of the joints. However, there are different kinds of arthritis, including RA and osteoarthritis (OA). Although RA and OA both affect the joints, they are very different forms of the same broader condition. RA is a ROS-mediated autoimmune disorder, while OA, the most common form of arthritis, is primarily a degenerative joint disorder^32^. Thus, we further examined p22phox interaction with Rubicon by comparing it between RA and OA patients. p22phox and Rubicon were more highly expressed in RA tissue than in OA tissue (Fig. 1d, left and right panels and Supplemental Fig. 2). The expression and colocalization of p22phox and Rubicon were markedly increased and there was strong positivity in the synoviocytes of RA patients, but not in OA patients (Fig. 1d, middle panel). These results show that p22phox associated with Rubicon in synoviocytes is clinically significant for both human RA and mouse CIA model.

### Rubicon gene expression affects RA mouse mortality

**Figure 2:**
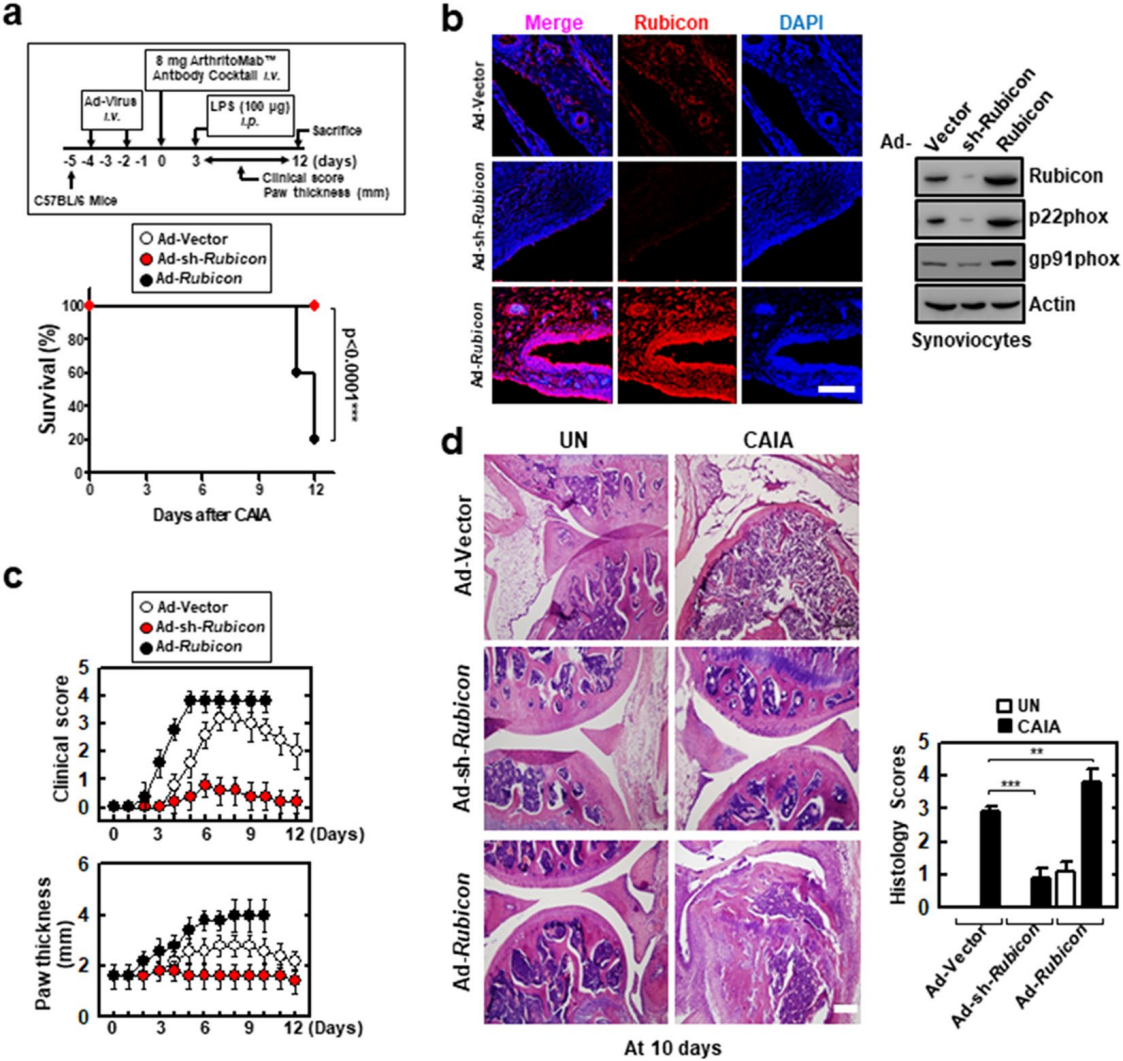
Alteration of Rubicon gene expression affects CAIA mice mortality. (**a**) Schematic of the collagen antibody-induced arthritis (CAIA) model (upper). At 48 hr post-injection with Ad-GFP, Ad-shRubicon, or Ad-Rubicon (1 × 10^13^ pfu/kg), twice intravenously via the tail vein, CAIA mice model was established. The survival of CAIA mice was monitored for 12 days and mortality was measured for n = 10 mice per group (lower). Statistical differences, as compared to the Ad-GFP-injected mice, are indicated (log-rank test). (**b**) Immune-stained with αRubicon or DAPI for Rubicon gene expression (left). IB with αRubicon, αp22phox, or αActin (right). Scale bars: 100 μm. (**c**) Clinical arthritis score and swelling of paws. Data shown are the means ± SD of three experiments. (**d**) Representative H&E staining of the ankle joints of each group determined at 9 days of CAIA (left). Scale bars: 200 μm. Histopathology scores (right) from ten mice per group. Statistical significance was determined by two-way analysis of variance (ANOVA) with Tukey’s posttest; ***P < 0.001 compared with Ad-Vector (**a**). Data shown are the means ± SD of three independent experiments (**c**). UN, untreated.

To assess whether the depletion or expression of Rubicon affects mice with *in vivo* CAIA, recombinant adenoviruses (Ad-vector, Ad-shRubicon, and Ad-Rubicon) were injected intravenously via the tail vein and then mice were challenged with CAIA for 2 days (Fig. 2a, top). Mice transduced with Ad-vector or Ad-shRubicon survived for more than 12 days. However, mice transduced with Ad-Rubicon showed significantly hastened mortality (median survival, 11 days) and a decreased survival rate (20% survival) (Fig. 2a, bottom). Rubicon genes knock-down and overexpression was confirmed by immunofluorescent staining and by Western blot in protein levels (Fig. 2b).

Correlated with the survival rate, arthritic clinical scores and paw swelling were markedly increased in Rubicon-expressing mice and decreased in Rubicon-depleted mice compared with the levels in mice transduced with Ad-Vector (Fig. 2c). Furthermore, histopathological evaluation of hind paws showed greater synoviocyte hyperplasia, bone erosion, and cartilage destruction in the joints of Rubicon-expressing mice than in the joints of mice transduced with Ad-vector (Fig. 2d). These results unambiguously show that pathophysiology is substantially affected by the level of Rubicon expression in mouse model for RA.

### TIPTP (p22 inhibitor) robustly suppresses p22phox-Rubicon interaction and ROS-mediated inflammation

**Figure 3:**
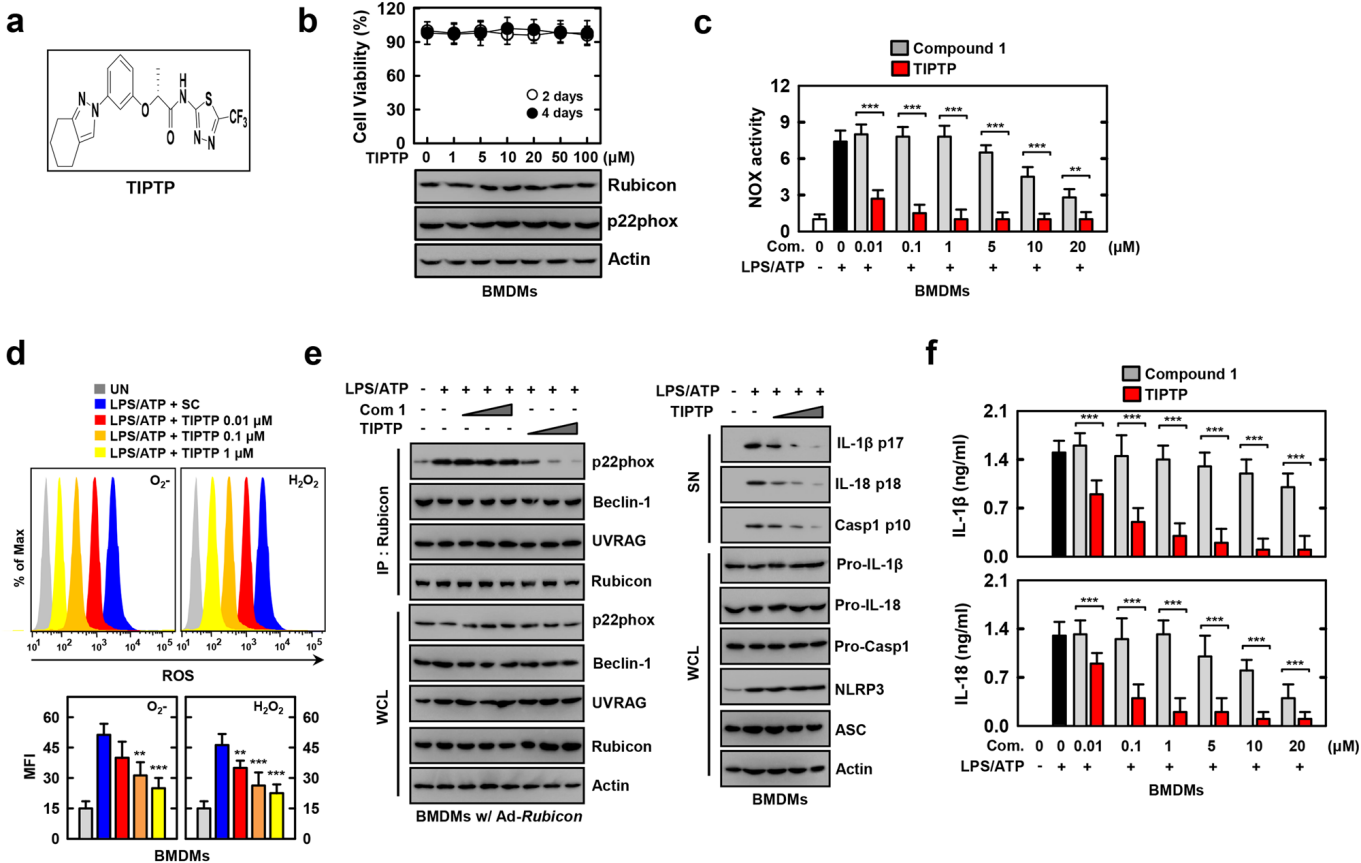
TIPTP robustly suppresses LPS/ATP-mediated Rubicon-p22phox interaction and ROS-mediated inflammation. (**a**) Structure of the TIPTP. (**b**) MTT assay for cell viability. BMDMs were incubated with TIPTP for the indicated times at the indicated TIPTP concentrations (upper). WCLs were used for IB with α22phox, αRubicon, or αActin (lower). (**c**–**f**) LPS (100 ng/ml)-primed BMDMs were treated with compounds for 1 h, and then activated with ATP (1 mM) for 30 min. (**c**) NADPH oxidase activity. (**d**) FACS analysis for superoxide and hydrogen peroxide (upper). Quantitative analysis of mean fluorescence intensities of ROS (lower). (**e**) BMDMs were incubated with increasing concentrations of compound 1 and TIPTP (com 2) (***). IP with αRubicon, followed by IB with αp22phox, αBeclin-1, αUVRAG, αRubicon. WCLs were used for IB with αp22phox, αBeclin-1, αUVRAG, αRubicon, or αActin (left). IB analysis of IL-1β p17, IL-18 p18, or caspase-1 p10 in supernatants (SN), and pro-IL-1β, pro-IL-18, or pro-caspase-1 in WCL, with αActin as a loading control (right). (**f**) Culture supernatants were harvested and analyzed for cytokines by ELISA. The data are representative of three independent experiments with similar results (**b** and **e**). Data shown are the means ± SD of three experiments (**b**–**d** and **f**). Statistical analysis was done using the Student’s *t*-test with Bonferroni adjustment (**P < 0.01; ***P < 0.001) compared with LPS/ATP alone (**c,d** and **f**). UN, untreated.

Currently available therapies are limited by toxicity issues. Against this background, we have developed a new propenamide, designated **TIPTP**, that inhibits the p22phox–Rubicon interaction. Initially, we synthesized a series of achiral or racemic (tetrahydroindazolyl)phenoxy propanamides containing pyridine or thiadiazole based on previously reported compounds. All screenings were repeated three times, and the compounds that showed reproducible inhibitory activity against p22phox–Rubicon interaction were primarily selected. Modifying the structure of these compounds, we further synthesized the corresponding propanamide derivatives as enantiomerically pure forms and evaluated them in an *in vitro* context. Based on the results of these assays, 2-(tetrahydroindazolyl)phenoxy-*N-(*thiadiazolyl)propanamide **2** (**TIPTP**, M.W. 437.44: Fig. 3a and Supplemental Figs. 3 and 4) was the most potent p22 inhibitor and was chosen for further investigation.

First, we measured the cytotoxicity of TIPTP to rule out the possibility that its inhibitory effect is derived from the inhibition of cell viability. We used macrophages for p22phox/gp91phox-Rubicon interaction and development of inhibitors. In particular, synovial fluid containing synovial cells is difficult to study because it is mixed with fibroblast-like and macrophage-like synovial cells. TIPTP did not induce any significant change in the cell viability of BMDMs after incubation with different concentrations (Fig. 3b, top). Furthermore, TIPTP did not affect the expression of p22phox or Rubicon proteins in BMDMs (Fig. 3b, bottom). Next, we tested whether TIPTP had N8 peptide-mimetic pharmacological (compound **1**) and biological profiles^23^. Consistent with the activity of compound **1**, TIPTP inhibited p22phox–Rubicon interaction (Supplemental Fig. 5). NOX activity (Fig. 3c and Supplemental Fig. 5b), ROS (O_2_^−^ and H_2_O_2_) (Fig. 3d), and inflammation (Supplemental Fig. 5b) generation were inhibited in a dose-dependent manner. Remarkably, TIPTP had an IC_50_ of 0.1 μM) which was a 100-fold improvement in IC_50_ compared with that of compound **1** which had an IC_50_ of 10 μM (Supplemental Fig. 5a). Furthermore, interactions of p22phox with Rubicon in Rubicon-expressing BMDMs or Raw464.7 cells were suppressed by pretreatment with TIPTP for 1 h prior to stimulation with TLR4/LPS or NLRP3/LPS + ATP (Fig. 3e, left panel and Supplemental Fig. 5c).

NLRP3 inflammasome activation contributes to the pathogenesis of RA and components of the NLRP3 inflammasome have been found to be expressed in the synovia of RA patients^22,33^. We found that TIPTP significantly decreased caspase-1 activation, and IL-1β and IL-18 maturation in response to NLRP3 inflammasome-activating stimuli in BMDMs (Fig. 3e, right panel and 3f). However, TIPTP inhibited p22phox–Rubicon interaction, but not NLRP3 inflammasome complex assembly (Supplemental Fig. 5b and d). Furthermore, Nox2ds-TAT is a chimeric 18-amino acid peptide that has been shown to inhibit NOX activity *in vivo* and *in vitro*^34,35^ to selectively inhibit the interaction between NOX2 and p47phox^36^, but not p22phox and Rubicon. In Supplemental Fig. 5E, LPS-induced NOX activity was inhibited by Nox2ds-TAT peptide in dose-dependent manner. However, this result is not due to the inhibition of p22phox and Rubicon interactions. These findings indicate that TIPTP acts as a selective and potent immunomodulatory agent by inhibiting p22phox-mediated NADHP oxidase complex assembly and inflammation.

### TIPTP (p22 inhibitor) shows reliable biochemical properties and pharmacokinetic profile

We next examined the biochemical stability and pharmacokinetic parameters of TIPTP before assessing its therapeutic potential *in vivo*. As shown in Table 1, TIPTP displayed excellent plasma stability, showing 97% retention (at 30 min) and 94% retention (at 2 h) after exposure to human plasma and it was easily metabolized in human hepatic microsomes. We also examined the inhibitory activity of TIPTP against three important CYP isozymes to evaluate the possibility of its drug–drug interaction (DDI). The results indicated that the effect of TIPTP on the CYP2D6 isozyme was negligible and the enzymatic activity of the other two CYP isozymes was not significantly decreased by TIPTP (Table 1). Therefore, TIPTP might be metabolized by multiple CYP enzymes to reduce its potential DDI.

**Table 1:** The results of *in vitro* plasma stability, microsomal stability, and CYP inhibition.

	M.W.	Human plasma stability (% remaining after 30 min and 2 h)	Human liver microsomal stability (% remaining after 30 min)	CYP (% of control activity @ 10 μM)		
				3A4	2D6	1A2
**TIPTP (2)**	437.44	97.4 and 93.8	4.4	76.6	98.2	45.7

Pharmacokinetic studies of TIPTP upon intraperitoneal and intravenous (IV) administration in male ICR mice were performed, the results of which are presented in Table 2. On the basis of the AUC values upon both types of administration, TIPTP was well tolerated in mouse plasma and maintained in the blood at a high concentration. Following IV administration, TIPTP was appropriately distributed throughout the blood and tissues (V_ss_ = 127.94 ml/kg). The clearance value upon IV administration confirmed that TIPTP was eliminated from systemic circulation at a moderate rate. The absolute bioavailability (*F*,%) of TIPTP in mice was considerably high after intraperitoneal administration (75.42%), which suggested that it was significantly absorbed through the peritoneum to enter the systemic circulation.

**Table 2:** Mean (±SD^a^) pharmacokinetic parameters in mouse plasma following intraperitoneal (n = 5, 1 mg/kg) and intravenous (n = 3, 0.5 mg/kg) administration of **TIPTP (2)** to male ICR mouse.

	IP	IV
AUC_last_ (ng min/ml)	15054.20 ± 3408.12	9980.68 ± 446.21
AUC_0-∞_ (ng min/ml)	15092.98 ± 3407.52	10036.03 ± 450.94
*C*_max_ (ng/ml)	5590.00 ± 1427.01	—
*T*_max_ (h)	0.23 ± 0.04	—
*T*_1/2_ (h)	2.91 ± 0.14	3.67 ± 0.39
CL (ml/h/kg)	—	49.89 ± 2.30
*V*_ss_ (ml/kg)	—	127.94 ± 24.57
MRT_inf_ (h)	3.42 ± 0.43	2.57 ± 0.52
*F*^b^ (%)	75.42 ± 17.07	—

AUC_0−∞_, total area under the plasma concentration−time curve from time zero to time infinity; AUC_last_, total area under the plasma concentration−time curve from time zero to last measured time; *C*_max_, peak plasma concentration; *T*_max_, time to reach *C*_max_; CL, time−averaged total body clearance; MR_Tinf_, mean residence time; V_ss_, apparent volume of distribution at steady state.

^a^SD: Standard deviations.

^b^*F* was calculated using AUC_last_.

### TIPTP (p22 inhibitor) protects mice from rheumatoid arthritis

**Figure 4:**
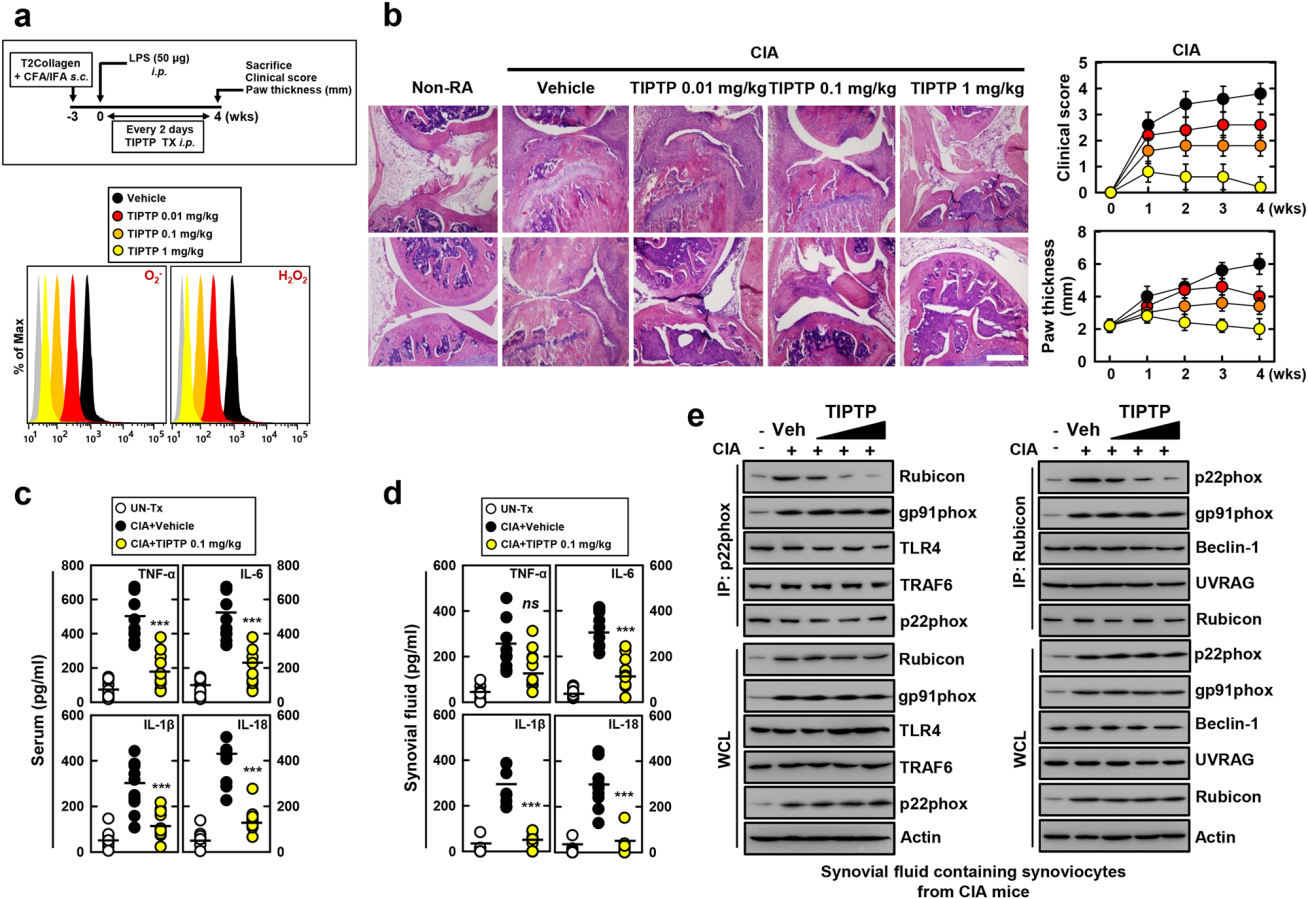
TIPTP protects mice from CIA mice. (**a**) Schematic of the CIA model treated with TIPTP (upper). FACS analysis for superoxide and hydrogen peroxide from synovial fluid containing synoviocytes from CIA (lower). (**b**) Representative H&E staining of the ankle joints of each group were determined at 4 wks of CIA (left). Clinical arthritis score and swelling of paws (right) from ten mice per group. Scale bars: 500 μm. Serum (**c**) and Synovial fluid (**d**) were harvested at 4 wks of CIA and analyzed for cytokines by ELISA. (**e**) Synovial fluid containing synoviocytes were used for IP with αRubicon or αp22phox, followed by IB with αRubicon, αp22phox, αgp91phox. αRubicon IPs were also blotted for αBeclin-1, or αUVRAG. αp22phox IPs were also blotted for αTLR4, and αTRAF6. WCLs were used for IB with αRubicon, αp22phox, αgp91phox, αTLR4, αTRAF6, αBeclin-1, αUVRAG, or αActin. The data are representative of three independent experiments with similar results (**a**,**b** and **e**). Data shown are the means ± SD of three experiments (**b**–**d**). Statistical analysis was done using the Student’s t-test with Bonferroni adjustment (***P < 0.001) compared with RA + Vehicle (**c** and **d**).

To determine whether TIPTP protects mice from ROS-mediated RA, we used a model of CIA (Fig. 4a, top) that resembles many clinical, histological, and immunological features of human RA^33,37^. First, we evaluated the effect of TIPTP on ROS production that was mechanistically linked to RA. TIPTP significantly attenuated the production of ROS by synovial tissues in a dose-dependent manner (Fig. 4a, bottom).

To further evaluate the effect of TIPTP on synovial pathology and cartilage destruction, the findings of these clinical assessments were confirmed by histological examination. Hematoxylin and eosin (H&E) staining of joints from CIA mice treated with vehicle revealed severe hyperplasia of synovia, pannus formation, and infiltration of inflammatory cells, whereas TIPTP treatment significantly reduced these alterations (Fig. 4b, immunohistochemistry). Semi-quantitative scoring of these mean clinical parameters, paw thickness, and histology confirmed that the severity of RA in TIPTP -treated CIA mice was markedly lower than in vehicle-treated CIA mice (Fig. 4b, right, and Supplemental Fig. 6a). Together, these findings suggest a protective role of TIPTP in the CIA arthritis mouse model.

ROS-producing NOX signaling is known to regulate the cytokine storm contributing to the pathogenesis of RA^6,20^. We therefore evaluated the effect of TIPTP on the production of inflammatory mediators that are mechanistically linked to RA. Consistent with the ROS data (Fig. 4a, bottom), serum and synovial fluid concentrations of the TNF-α, IL-6, IL-1β, and IL-18 proinflammatory cytokines were significantly attenuated (Fig. 4c,d). In addition, caspase-1 activation, and IL-1β and IL-18 maturation levels in synovial fluid were significantly attenuated in TIPTP-treated CIA mice (Supplemental Fig. 6b). Furthermore, we tested whether this compound has pharmacological activity *in vivo. In vivo* detection of phagocytosis-related target protein binding profiles and autophagy activity might be important for evaluating TIPTP in the search for therapeutic drugs to treat inflammatory disease^18,23^. Consistent with the *in vitro* data (Fig. 3e and Supplemental Fig. 5c), treatment with TIPTP markedly decreased the binding of p22phox with Rubicon protein in synoviocytes from CIA mice (Fig. 4e). This demonstrated that TIPTP specifically blocked p22phox–Rubicon interaction without affecting Rubicon–Beclin1–UVRAG or NLRP3 inflammasome complex interaction. Taken together, these findings suggest that TIPTP has therapeutic potential to ameliorate severity in the CIA mouse model.

### TIPTP (p22 inhibitor) protects mice from severe arthritis

**Figure 5:**
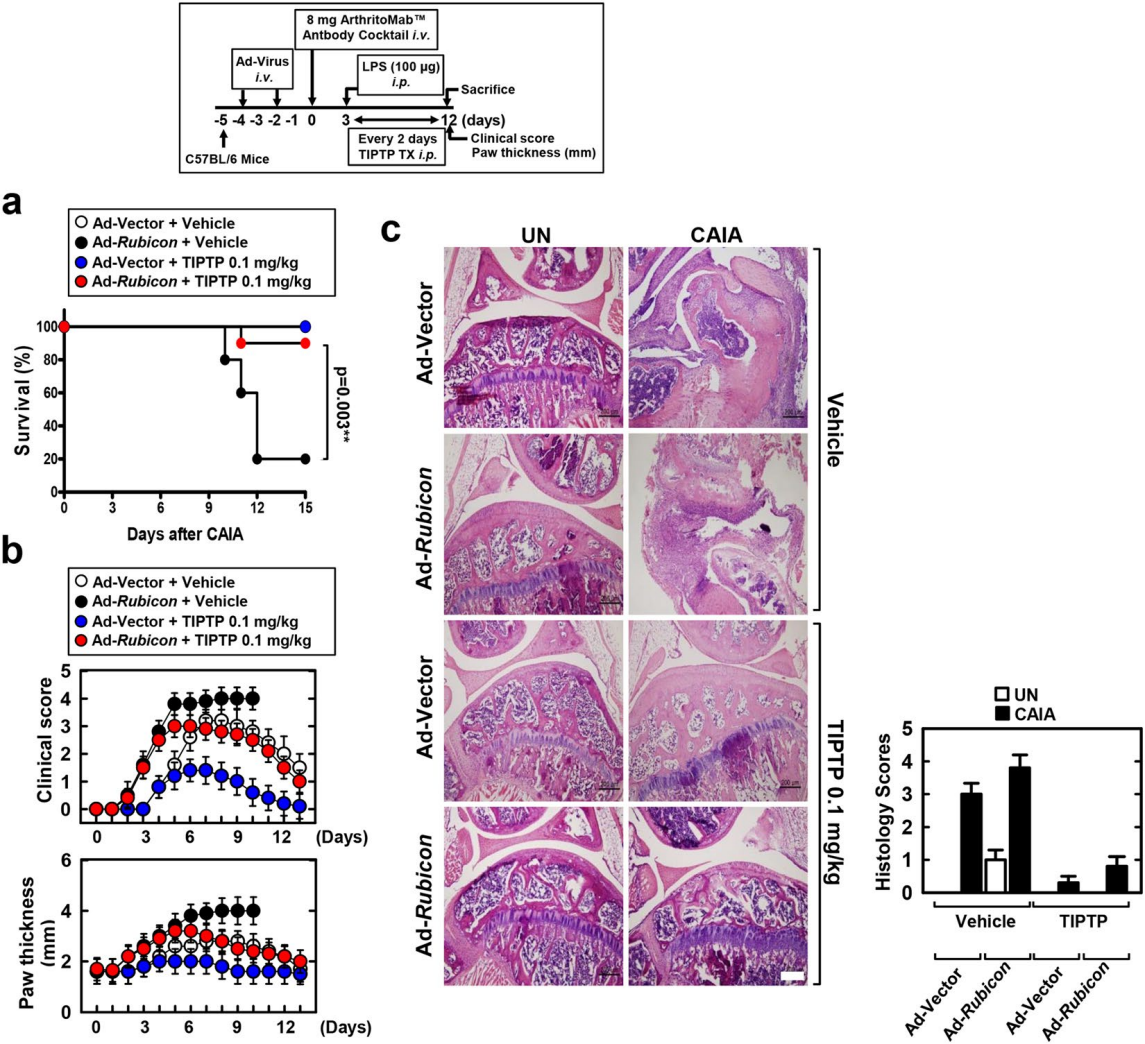
TIPTP protects Rubicon-expressed CAIA mice mortality. Schematic of the CAIA model treated with TIPTP (upper). (**a**) The survival of CAIA mice was monitored for 15 days and mortality was measured for n = 10 mice per group. Statistical differences, as compared to the Ad-Rubicon + Vehicle, are indicated (log-rank test). (**b**) Clinical arthritis score and swelling of paws, or (**c**) representative H&E staining of the ankle joints of each group were determined at 9 days of CAIA (left). Histopathology scores (right) from ten mice per group. Scale bars: 200 μm. The data are representative of three independent experiments with similar results. Statistical significance was determined by two-way analysis of variance (ANOVA) with Tukey’s posttest; **P < 0.01 compared with Ad-Vector+Vehicle (**a**). Data shown are the means ± SD of three experiments (**b** and **c**). UN, untreated.

We have developed a severe mouse model for RA, CAIA mice transduced with Ad-Rubicon (Fig. 2, top). Therefore, we further investigated whether TIPTP protects Rubicon-overexpressing CAIA mice. First, we tested the therapeutic efficacy of TIPTP against CAIA-induced mortality in mice. CAIA mice were post-treated with TIPTP every 2 days via intraperitoneal injection; notably, CAIA mice transduced with Ad-Rubicon showed median survival of 11 days (20% survival), but equivalent mice treated with TIPTP showed a significant delay in mortality and an increased survival rate (90% survival) (Fig. 5a, bottom). Consistent with the mortality data, arthritic clinical scores, paw swelling, and histopathological evaluation were markedly decreased in both Rubicon-expressing and vector CAIA mice treated with TIPTP compared with the levels in mice treated with vehicle (Fig. 5b,c). Taken together, these results unambiguously show that TIPTP has therapeutic potential to ameliorate arthritis.

### TIPTP (p22 inhibitor) is active *ex vivo* for cells from healthy humans or RA patients

**Figure 6:**
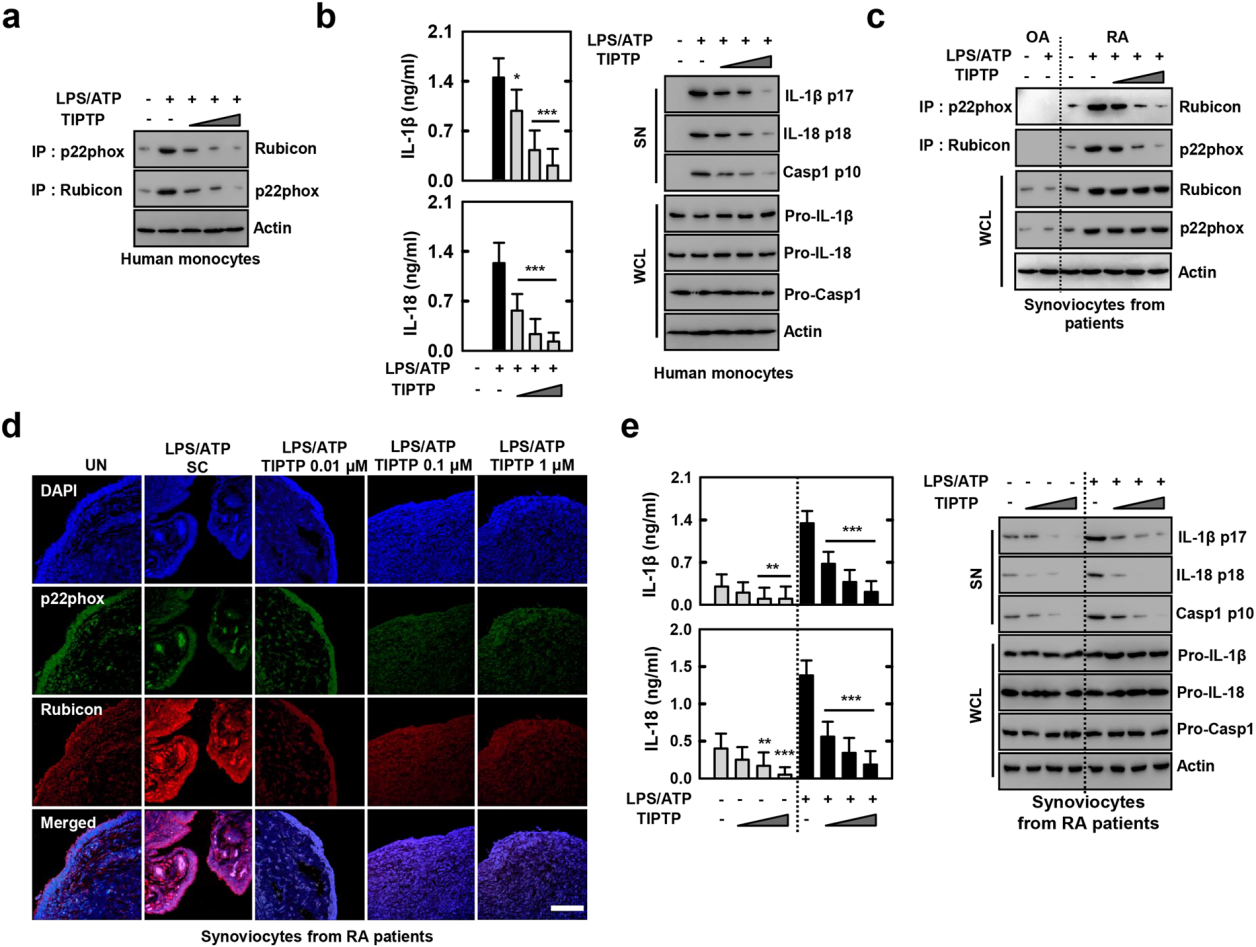
TIPTP is active for cells from healthy humans or patients with RA. (**a** and **b**) LPS-primed human monocytes were treated with TIPTP (0.01, 0.1, 1 μM) for 30 min, and then activated with ATP for the 30 min. (**a**) IP with αp22phox or αRubicon, followed by IB with αRubicon or αp22phox. Loading control was used for IB with αActin. (**b**) Culture supernatants were harvested and analyzed for cytokines by ELISA (left). IB analysis of IL-1β p17, IL-18 p18, or caspase-1 p10 in SN, and pro-IL-1β, pro-IL-18, or pro-caspase-1 in WCL, with αActin as a loading control (right). (**c**) LPS-primed synoviocytes from patients with OA or RA were treated with TIPTP, and then activated with ATP. IP with αp22phox or αRubicon, followed by IB with αRubicon or αp22phox. Loading control was used for IB with αActin. (**d**) Synoviocytes form RA patients were stained with αp22phox (Alexa Fluor 488; green) and αRubicon (Cy3; red). Nuclei were counterstained with DAPI. Cells were visualized by confocal microscopy. Scale bars: 100 μm. (**e**) Culture supernatants were harvested and analyzed for cytokines by ELISA (left). IB analysis of IL-1β p17, IL-18 p18, or caspase-1 p10 in SN, and pro-IL-1β, pro-IL-18, or pro-caspase-1 in WCL, with αActin as a loading control (right). The data are representative of three independent experiments with similar results (**a**–**e**). Data from one of sixteen RA or ten OA patients are shown (**c**). Data shown are the means ± SD of three experiments and Statistical analysis was done using the Student’s *t*-test with Bonferroni adjustment (**P < 0.01; ***P < 0.001) compared with LPS/ATP (**b** and **e**). SN: supernatant.

We further investigated whether TIPTP was effective for human cells. First, we found that in a dose-dependent manner TIPTP inhibited p22phox–Rubicon interaction in normal human monocytes treated with LPS/ATP (Fig. 6a). In a dose-dependent manner, TIPTP also inhibited IL-1β and IL-18 proinflammatory cytokines, caspase-1 activation, and IL-1β and IL-18 maturation levels in human blood-derived monocytes treated with LPS/ATP (Fig. 6b). These results suggest that TIPTP can prevent ROS-mediated inflammation in human cells.

We then tested whether TIPTP affected pre-activated ROS-producing NOX and Rubicon on cells from RA patients with abnormal ROS activation^16,17^. First, we found marked increases in the levels of p22phox and Rubicon in RA patient cells, and that TIPTP could efficiently inhibit p22phox–Rubicon interaction in RA patient cells from WB and confocal images (Fig. 6c,d). Notably, RA patients-derived synoviocytes were NOX1 to NOX5 expressed (Supplemental Fig. 7). The difference in expression pattern between mouse and human seems to be the specificity of species and the amount of cells (protein) used in the experiment. However, in Supplemental Fig. 7, consistent with the findings shown in Fig. 1c and Supplemental Fig. 1, p22phox interact with NOX2, NOX4 or NOX5 (human only). Furthermore, consistent with the findings shown in Fig. 4e and Supplemental Fig. 5c, p22phox-Rubicon interaction inhibited by TIPTP *vice versa*. The p22phox-NOX2, NOX4, or NOX5 interaction was not inhibit by TIPTP. Taken together, TIPTP (p22phox inhibitor) was p22phox-Rubicon interaction specific inhibitor in synoviocytes. Furthermore, as expected, when freshly isolated synoviocytes from synovial fluid cells from a patient with RA were incubated without the stimulation of NLRP3 agonists, IL-1β and IL-18 secretion and caspase-1 processing could be detected in the culture supernatants (Fig. 6e). However, when these cells were incubated in the presence of TIPTP, the caspase-1 activation, and IL-1β and IL-18 production were inhibited in a dose-dependent manner (Fig. 6e). These results indicate that TIPTP can suppress the pre-activated ROS-producing NOX and Rubicon from RA patients and suggest that TIPTP might be used to control ROS-driven diseases in a clinical context.

## Discussion

The central finding of this study is the existence of a potent, selective, and direct inhibitor of p22phox with enhances inhibitory activity against ROS-producing NOX (p22phox) and Rubicon interaction (an interaction that leads to ROS production from RA in mice *in vivo* and in human cells *ex vivo*). Specifically, we (1) found that *in vivo* p22phox interacts with Rubicon and is necessary for increased ROS-mediated RA pathogenesis; (2) revealed that the expression and colocalization of p22phox and Rubicon were markedly increased and strongly positive in synoviocytes of RA patients and CIA mice which suggest that this interaction may be clinically significant; (3) Unlike in macrophages, Rubicon binds to the free form of p22phox thus does not regulated p22phox-gp91phox expression by Rubicon in synoviocytes; (4) developed a new aryl propenamide derivative, TIPTP, that consists of tetrahydroindazole and thiadiazole, and is a p22phox inhibitor that shows considerably improved potency and selectivity as compared to our previous compound and inhibits p22phox-mediated NOX complex assembly; and (5) showed that the direct targeting of p22hox–Rubicon by TIPTP could inhibit ROS-mediated NLRP3 inflammasomes *in vivo* and had remarkable therapeutic effects *ex vivo* in monocytes from healthy individuals or synovial fluid cells from RA patients and mouse models for RA. This above approach likely provides a novel and amenable therapeutic avenue for the treatment of inflammatory diseases induced by ROS, such as RA and others in patients, with inflammation that is considered “out-of-control”, due to which fatal outcomes are often encountered.

The mouse model is the most commonly tool to verify the efficacy of candidate therapies on human diseases^38,39^. Mouse models provide insight into the immune responses mechanisms of many diseases. However, the results of many animal studies have not been validated in human clinical trials^40^. Therefore, the results of this *in vivo* mouse model should be interpreted with caution to apply human disease. A variety of mouse models of human RA is established such as CIA, CAIA, tumor necrosis factor-transgenic mice, streptococcal cell wall-induced arthritis, proteoglycan-induced arthritis, and K/BxN-transgenic nice with specific features are available to investigate unique arthritic conditions^41^. To overcome the limitations of these animal models, we validated the effects of p22phox inhibitor in both CIA and CAIA, which have been used most commonly as models for RA. This study demonstrates that a TIPTP shows improved effects in both of these mouse models for RA. In addition to mouse model, TIPTP is found to specifically inhibit p22phox Rubicon interactions in RA patient cells but not in OA patient cells. Taken together, these results strongly support the novel p22phox, TIPTP, as possible therapeutic substances for RA.

Even though certain compounds were found to show good attenuating activity against the NLRP3 inflammasome-mediated RA as evidenced in some animal models, their clinical use was limited because of several nonspecific effects. The possibility of sulforaphane’s inhibition of AIM2 or NLRC4 inflammasomes compromising their role in host defense was indicated before^42^. The extensive anti-inflammatory activity of various drugs including BAY 11–7082, INF39, sulforaphane, β-hydroxybutyrate, isoliquiritigenin and parthenolide^43–48^ indicates their potential side effects as immunosuppressive agents, which may enhance the possibility of infection. Inhibition of NLRP3 inflammasome activation could be achieved by different compounds by various mechanisms, and these include the flufenamic acid and mefenamic acid effects on chloride efflux, influence on potassium ion efflux by BHB, and the effects of MCC950 on chloride and other volume-regulated anion channels. Thus, these compounds act by targeting an upstream event in NLRP3 signaling and, therefore have other inevitable biological effects, even though their precise mechanism of action is not known^49–52^. Recently, it has been shown that a specific NLRP3 inflammasome inhibitor, CY-09, is able to directly inhibit NLRP3 and it was also shown that CY-09 exhibited strong therapeutic or preventive effects on mouse models of gout, type 2 diabetes and cryopyrin-associated autoinflammatory syndrome^53^. However, considering that ROS are the major mediators of RA pathogenesis, unless ROS are controlled, an effective RA treatment cannot be achieved. Therefore, inhibitors of NOX that forms ROS offers a better and more specific therapeutic target than inhibiting NLRP3, for RA treatment. Furthermore, recent paper show that Rubicon is one of the few known negative regulators of autophagy, it has been indicated in literature that Rubicon is known to increase with age, contributing to the decline in autophagy^54^. In this study, the patient cohorts with RA are approx. 60 years old. At this age, it would be expected that there would be an abundance of Rubicon present for the p22phox to interaction and co-localization. Therefore, TIPTP is expected to affect RA patients *ex vivo*.

In the present study, we describe a specific TIPTP that selectively alleviated production of ROS by directly targeting p22phox. We also observed that TIPTP displayed marked therapeutic effects on different models of RA in mice such as CIA and Rubicon-expressed CAIA. Thus, we describe a specific TIPTP, which has the therapeutic potential to treat RA diseases arising due to ROS. Several lines of evidence points toward the pathogenic role of NOX-derived ROS in RA^7,8,10,15,16,22^. Thus, elevated ROS levels and production along with oxidative stress are considered as potential biomarkers for RA in patients^16^. Signaling pathways of NF-kB and NLRP3 inflammasome and the downstream events, which are activated by ROS, regulate the synthesis of proinflammatory cytokines and their release, and thereby lead to bone resorption, inflammation of joints, and cartilage degeneration in animal models of arthritis and RA patients^6,20–22^. Thus, these results strongly support the view that selective targeting of NOX, the main producer of ROS is beneficial in treating RA. Molecular characterization of such signaling molecules may pave the way to the improvement of specific therapies to treat RA.

We previously reported an N-terminal eight-amino-acid N8 peptide derived from p22phox and mimetic compounds from *in silico* virtual screening that blocks Rubicon–p22phox interaction, profoundly suppressing ROS and inflammatory cytokine production; this in turn dramatically reduced the mortality associated with CLP-induced polymicrobial sepsis in mice^23^. By further developing this previously established compound, we developed a TIPTP (p22 inhibitor) that showed considerably improved potency and selectivity as an RA therapeutic. The TIPTP improved the problems with stability, bioavailability, and metabolic ability *in vivo*. Moreover, small molecule compounds have advantages over peptides and biological agents (monoclonal antibodies). These advantages include easy diffusion across the cell membrane, cost-effectiveness, potential to be administered orally, relatively easy titration of doses, and being mostly non-immunogenic^55^. Although biosimilars have been considered for the treatment of RA, the intrinsic differences between the biosimilars may lead to wide variations in their safety, clinical efficacy as well as immunogenicity^56^. Thus, switching to biosimilars may be viewed upon as an alternate option when changing the clinical management of RA treatment. In this connection, TIPTP likely offers an unique resource for the development of a selective RA therapeutic. In further studies, it is necessary to devise a lesion-specific therapeutics administration method to induce better RA therapeutic effects. Recent efforts have instead focused on the development of high-performance therapeutics delivery systems that delay RA progress and enhance therapeutics efficiency by improving drug pharmacokinetics and reducing toxicity-related side effects. In this regard, nanoparticle-based delivery systems hold multiple advantages such as high loading capacity, controlled drug release, and direct targeting to joint lesion^57,58^.

## Conclusions

Finally, the results of this study together with earlier studies, have implicated p22phox biology in the clinical manifestation of RA. On the basis of our results, we suggest that blocking the interaction between p22phox and Rubicon, thereby affecting the innate immunity-related machinery as a novel strategy to control RA. We also propose that TIPTP is a new type of immunomodulatory therapeutic agent, which acts by inhibiting ROS-producing NOX. Our results strongly support the idea that TIPTP has a great potential as a therapeutic against RA through the control of elevated inflammatory responses. Therefore, there is need to systematically evaluate the therapeutic as well as biological effects of the TIPTP, in the clinical setting. Besides, our observations also provide novel methods for discovering and designing the new drugs for treating inflammation.

## Supplementary information

SUPPLEMENTARY INFORMATION.
