# Supplementary material for: Identification of highly potent and selective inhibitor, TIPTP, of the p22phox-Rubicon axis as a therapeutic agent for rheumatoid arthritis
Source: Sci Rep. 2020 Mar 12;10:4570. doi: 10.1038/s41598-020-61630-x (PMC7067850; doi:10.1038/s41598-020-61630-x)
Supplement: Supplementary file 1 — SUPPLEMENTARY INFORMATION. [file 41598_2020_61630_MOESM1_ESM.docx]

**Supplementary Information**

**Identification of highly potent and selective inhibitor, TIPTP, of the p22phox-Rubicon axis as a therapeutic agent for rheumatoid arthritis**

Ye-Ram Kim^a,b,#^, Jae-Sung Kim^a,b,#^, Su-Jin Gu^c,#^, Sungsin Jo^d^, Sojin Kim^a^, Sun Young Kim^a,b^, Daeun Lee^a^, Kiseok Jang^e^, Hyunah Choo^f^, Tae-Hwan Kim^d^, Jae U. Jung^g^, Sun-Joon Min^c,*^, Chul-Su Yang^a,b,*^

Running title: Improving the selectivity of p22phox inhibits rheumatoid arthritis

^a^Department of Molecular & Life Science, Hanyang University, Ansan 15588, S. Korea; ^b^Department of Bionano Technology, Hanyang University, Seoul 04673, S. Korea; ^c^Department of Chemical & Molecular Engineering/Applied Chemistry, Ansan 15588, S. Korea; ^d^Hanyang University Hospital for Rheumatic Diseases, Seoul 04763, S. Korea; ^e^Department of Pathology, Hanyang University College of Medicine, Seoul 04763, S. Korea; ^f^Center for Neuro-Medicine, Brain Science Institute, Korea Institute of Science and Technology (KIST), Seoul 02792, S. Korea; ^g^Department of Molecular Microbiology and Immunology, Keck School of Medicine, University of Southern California, Los Angeles, CA, 90089, USA; ^#^These authors contributed equally to this work.

***Correspondence to: Chul-Su Yang**

Department of Molecular & Life Science,

College of Science and Convergence Technology, Hanyang University, Ansan 15588, S. Korea.

Phone: 82-31-400-5519. Fax: 82-31-436-8153.

E-mail: [chulsuyang@hanyang.ac.kr](mailto:chulsuyang@hanyang.ac.kr)

***Correspondence to: Sun-Joon Min**

Department of Chemical & Molecular Engineering/Applied Chemistry,

College of Science and Convergence Technology, Hanyang University, Ansan 15588, S. Korea.

Phone: 82-31-400-5502. Fax: 82-31-400-5457.

E-mail: [sjmin@hanyang.ac.kr](mailto:sjmin@hanyang.ac.kr)


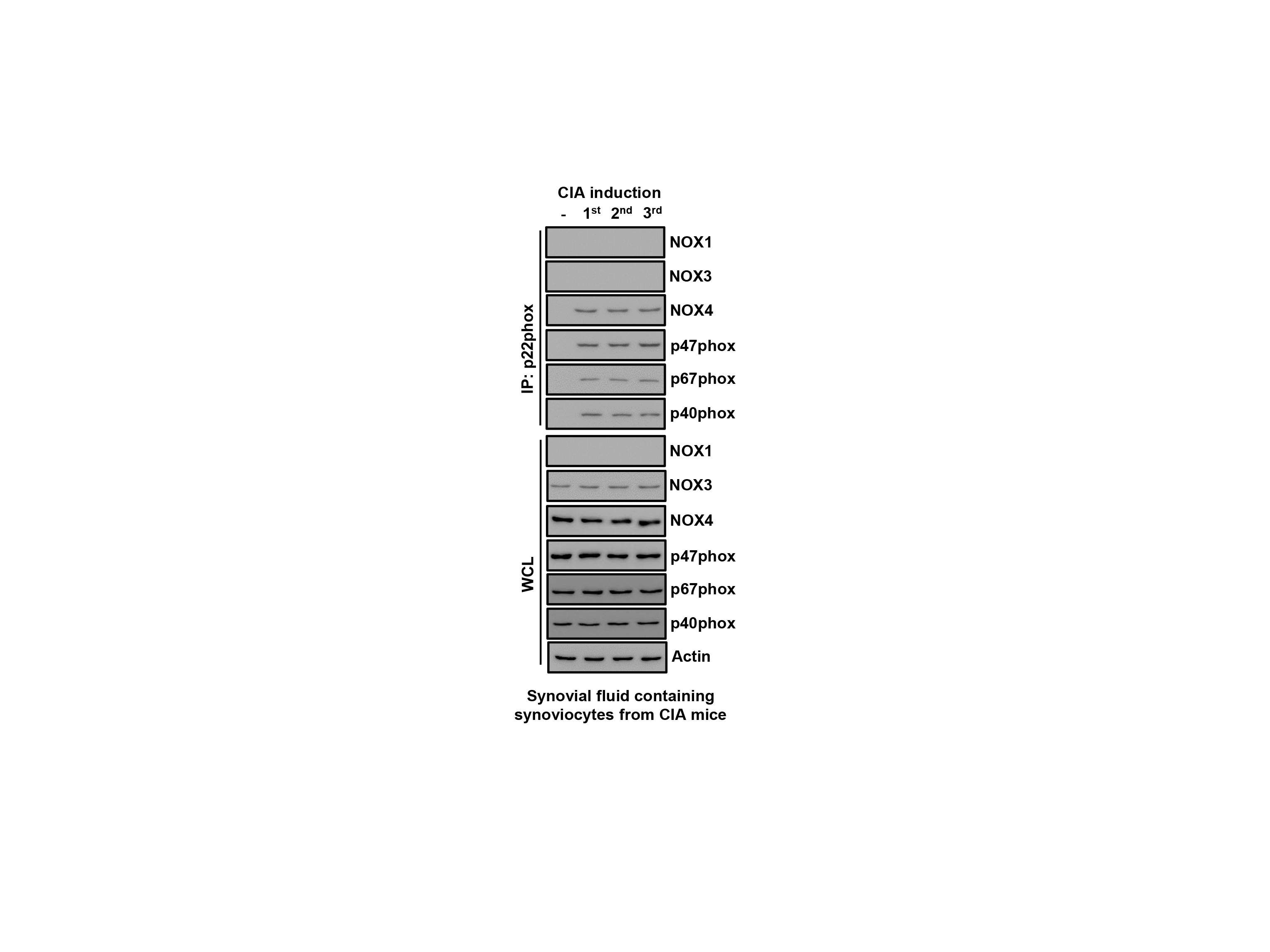


**Figure S1. p22phox interaction with NOXs in CIA mice**

Synovial fluid containing synoviocytes from CIA mice for the indicated times, followed by IP with αp22phox, followed by IB with αNOX1, αNOX3, αNOX4, αp47phox, αp67phox, and αp40phox. WCLs were used for IB with αNOX1, αNOX3, αNOX4, αp47phox, αp67phox, and αp40phox. Actin Western blot was used as a loading control. The data are representative of five independent experiments with similar results.


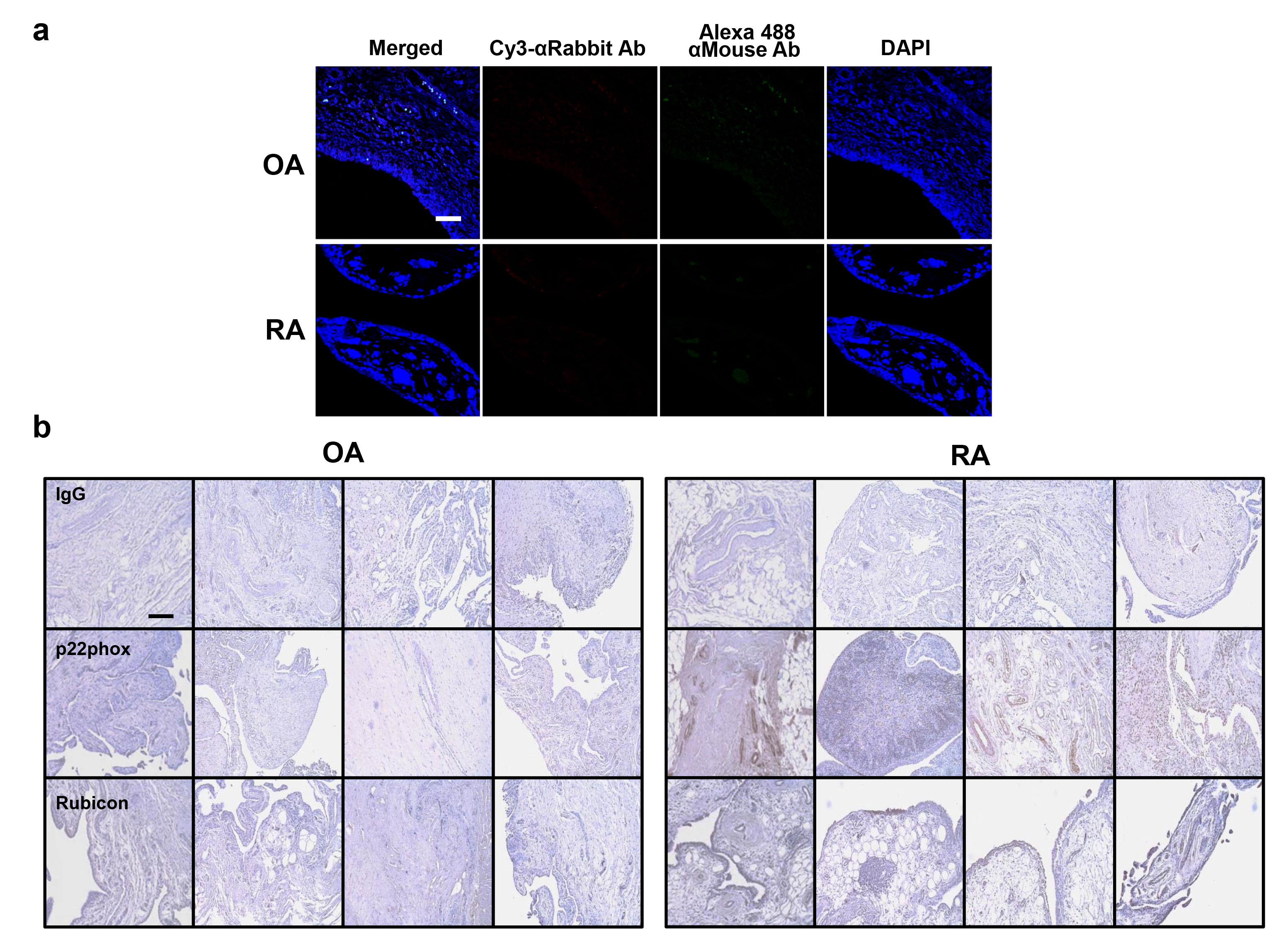


**Figure S2. Comparison of p22phox and Rubicon expression between OA and RA from patients.** (**a**) OA and RA cells were stained with Alexa Fluor 488-αMouse (green) and Cy3-αRabbit (red). Nuclei were counterstained with DAPI. Cells were visualized by confocal microscopy. (**b**) Representative photos for p22phox and Rubicon staining of ankle joint sections as described in **Supplemental Methods**. The data are representative of five independent experiments with similar results. Scale bar, 200 μm.

**
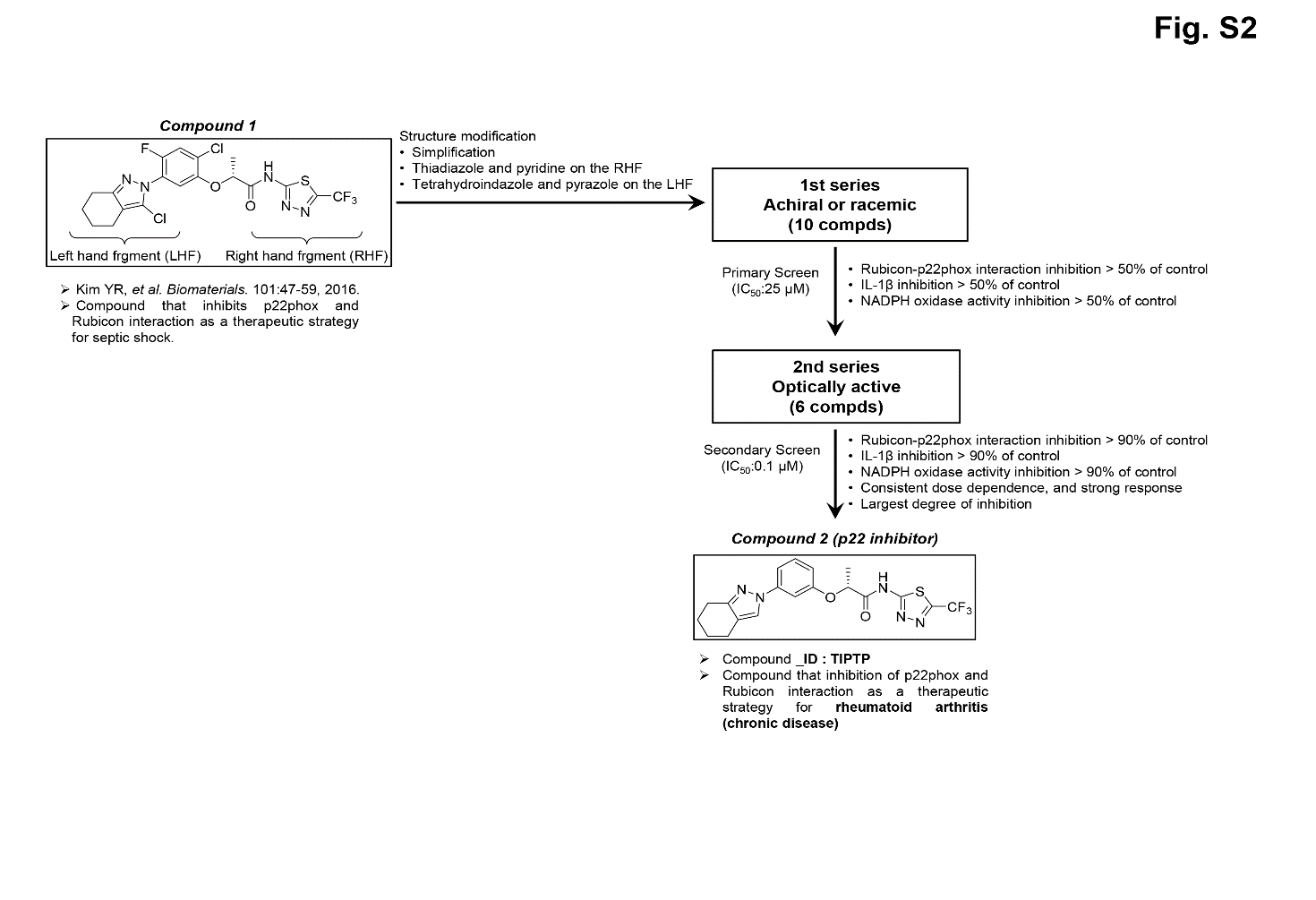
**

**Figure S3. Flow chart and summary of “hit” from primary and secondary screening**

Experimental procedures are described in **Supplemental Methods**.

**
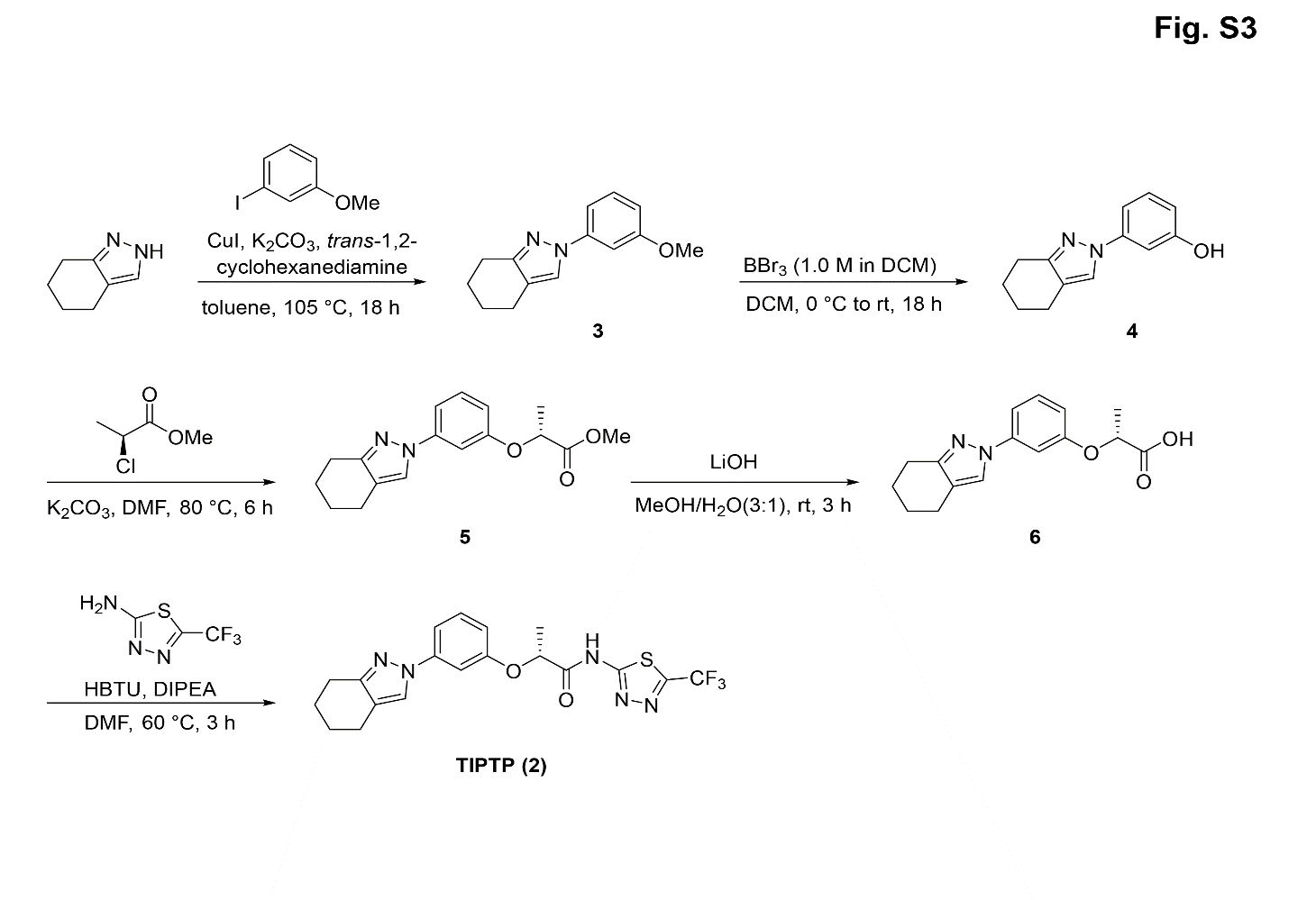
**

**Figure S4. Synthesis of 2-(tetrahydroindazolyl)phenoxy-*N*-(thiadiazolyl)propanamide (TIPTP).** Experimental procedures are described in **Supplemental Methods**.


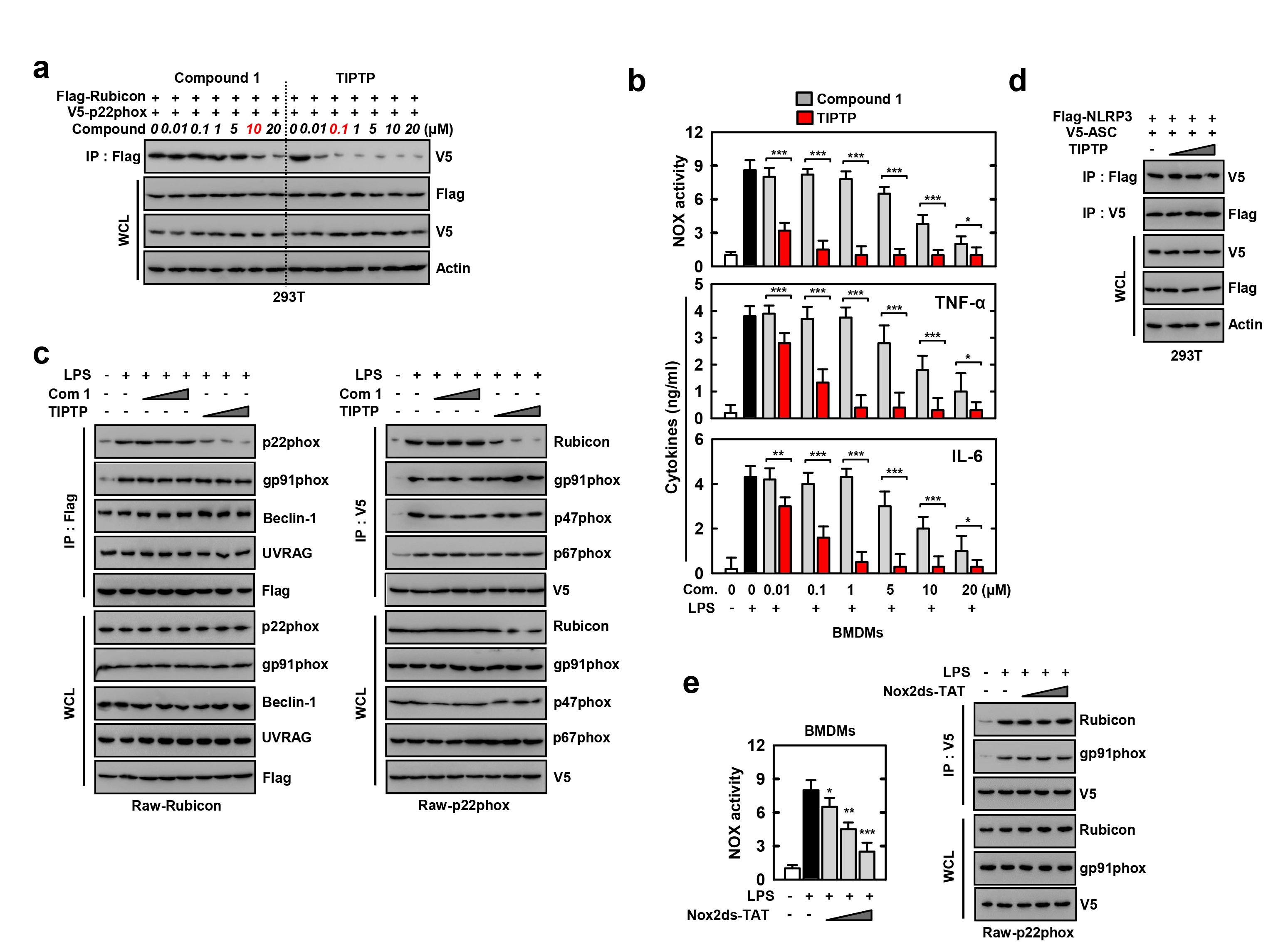


**Figure S5. Comparability of IC_50_ between compound 1 and 2 (TIPTP)**

(**a**) 293T cells were co-transfected with V5-p22phox and Flag-Rubicon. 12 h post transfection, cells were treated with compound 1 or 2 (0.01, 0.1, 1, 5, 10, 20 μM) for 24 h, and subjected to IP with αFlag, followed by IB with αV5. WCLs were used for IB with αFlag, αV5, or αActin. (**b**) NADPH oxidase activity and levels of cytokines by ELISA of BMDMs was stimulated with LPS for 30 min in the presence of compound 1 or 2 for 30 min or 18 h, respectively. Data shown are the means ± SD of three experiments. Significant differences (**P* < 0.05; ***P* < 0.01; ****P* < 0.001) compared with compound 1. (**c**) Raw264.7 cells containing Rubicon or p22phox were pretreated with compound 1 or 2 (0.01, 0.1, 1 μM) for 30 min, and then stimulated with LPS (100 ng/ml) for the 30 min., followed by IP with αFlag or αV5, followed by IB with αp22phox, αgp91phox, αBeclin-1, or αUVRAG, αRubicon, αp47phox, or αgp67phox. WCLs were used for IB with αFlag, or αActin. (**d**) 293T cells were co-transfected with V5-ASC and Flag-NLRP3. After 12 h posttransfection, cells were treated with compound 2 (0.01, 0.1, 1 μM) for 24 h, and subjected to IP with αFlag or αV5, followed by IB with αV5 or αFlag. WCLs were used for IB with αFlag, αV5, or αActin. (**e**) NADPH oxidase activity and levels of cytokines by ELISA of BMDMs was stimulated with LPS for 30 min in the presence of Nox2ds-TAT (1, 5, 10 μM) (left). Raw264.7 cells containing p22phox were pretreated with Nox2ds-TAT for 30 min, and then stimulated with LPS (100 ng/ml) for the 30 min., followed by IP with αV5, followed by IB with αRubicon or αgp91phox. WCLs were used for IB with αV5, or αActin (right). The data are representative of three independent experiments with similar results (**a**, **c**, **d**, and **e**).

**
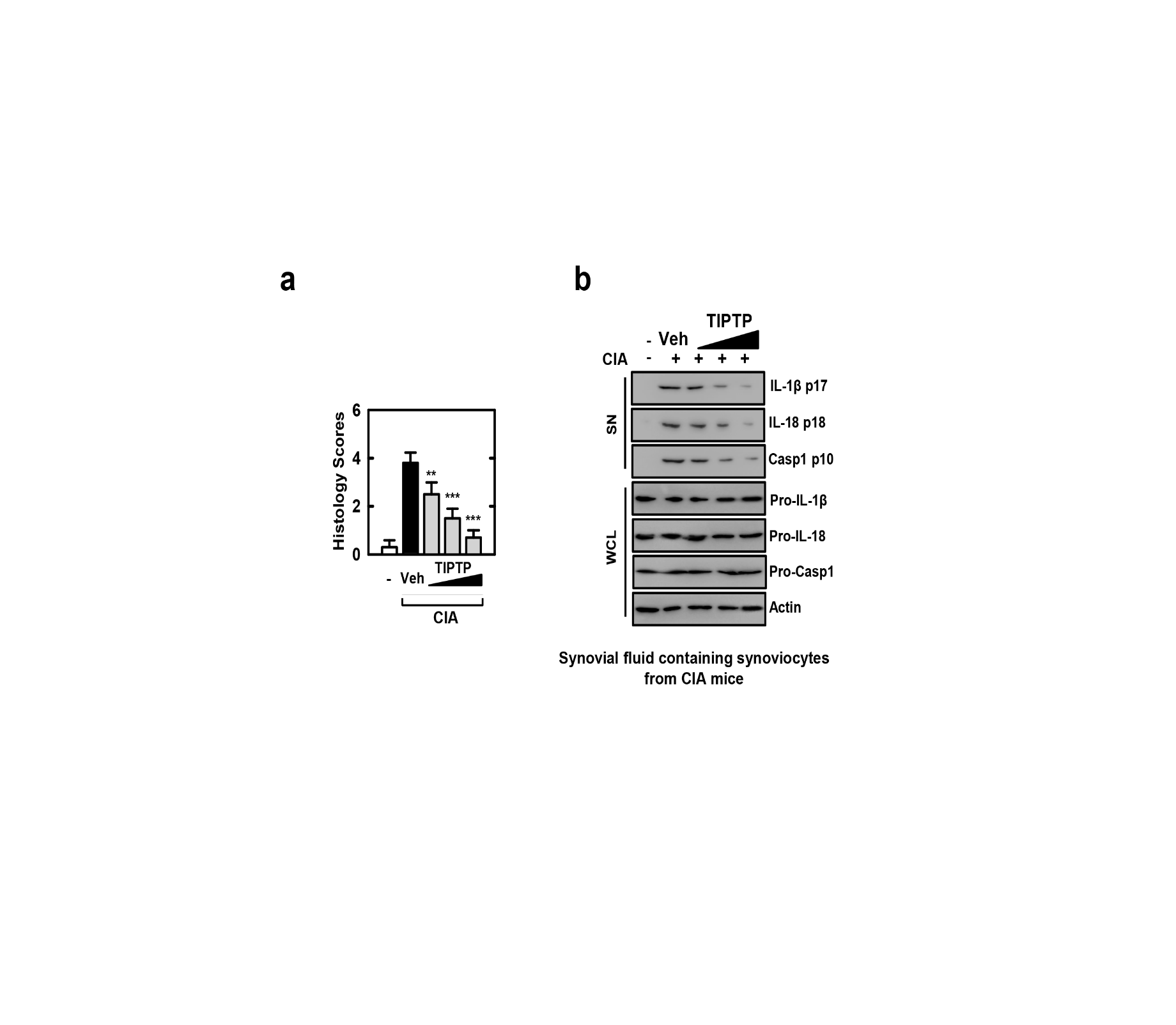
**

**Figure S6. The effects of TIPTP in CIA mice**

(**a**) Histopathology scores from ten mice per group. (**b**) Synovial fluid containing synoviocytes from patients with RA were used for IB analysis of IL-1β p17, IL-18 p18, or caspase-1 p10 in SN, and pro-IL-1β, pro-IL-18, or pro-caspase-1 in WCL, with αActin as a loading control. The data are representative of three independent experiments with similar results.

**
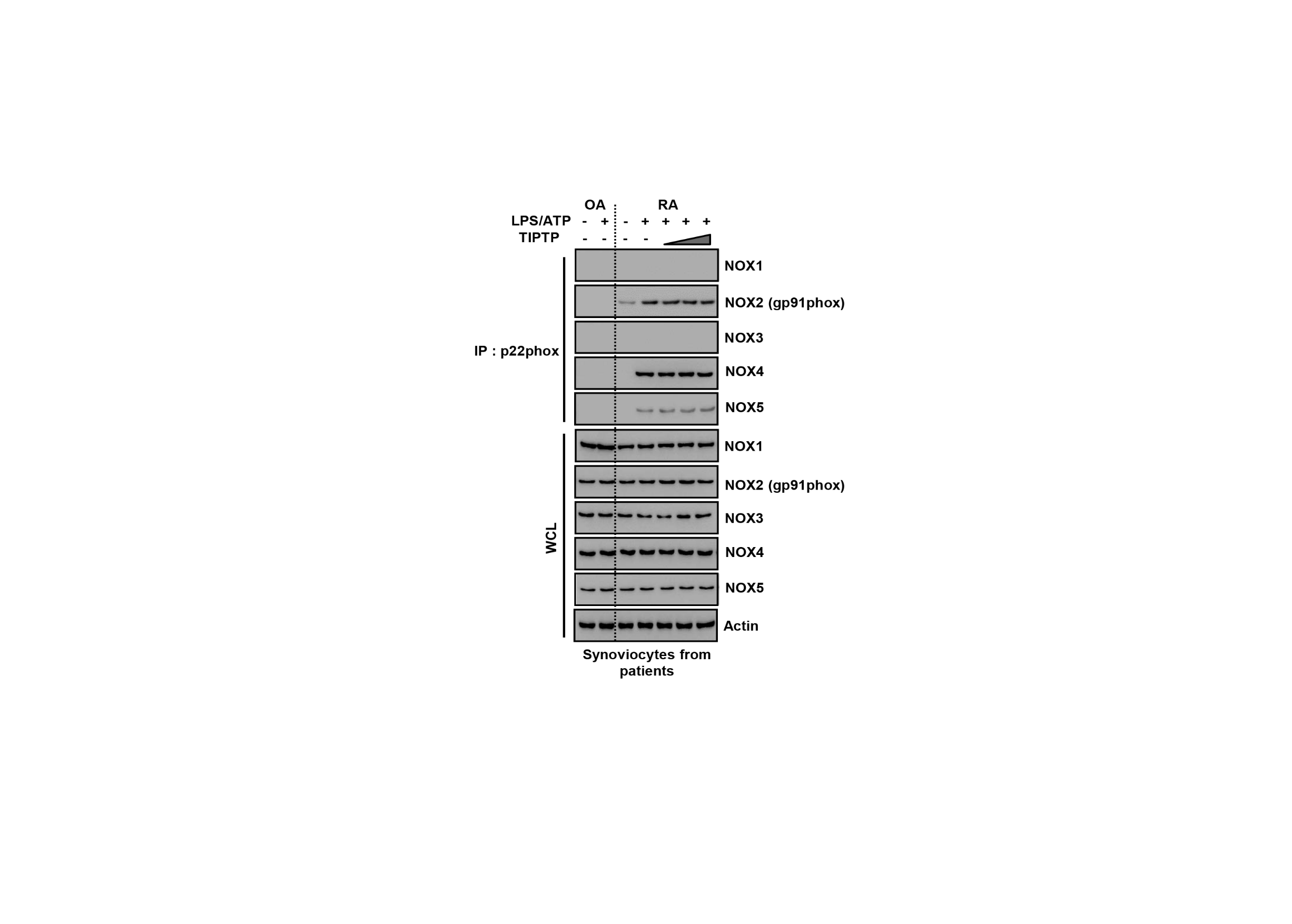
**

**Figure S7. The effects of TIPTP in CIA mice**

LPS-primed synoviocytes from patients with OA or RA were treated with TIPTP, and then activated with ATP. IP with αp22phox, followed by IB with αNOX1, αNOX2, αNOX3, αNOX4, and αNOX5. WCLs were used for IB with αNOX1, αNOX2, αNOX3, αNOX4, and αNOX5. Actin Western blot was used as a loading control. The data are representative of five independent experiments with similar results.


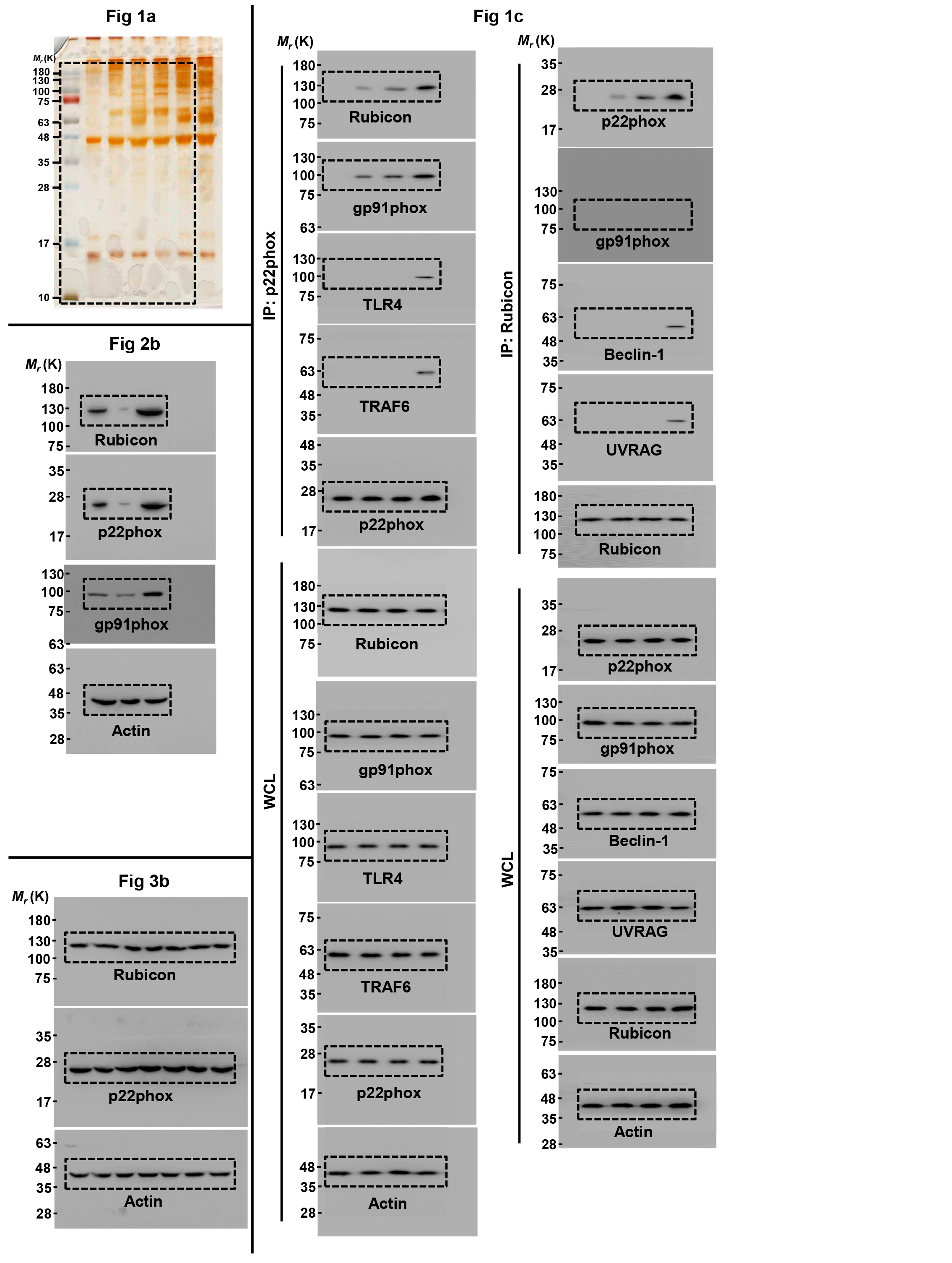


**Figure S8. Full-length western blots**

Full-length images of the blots presented in the **Fig. 1a**, **1c**, **2b** and **3b**.**
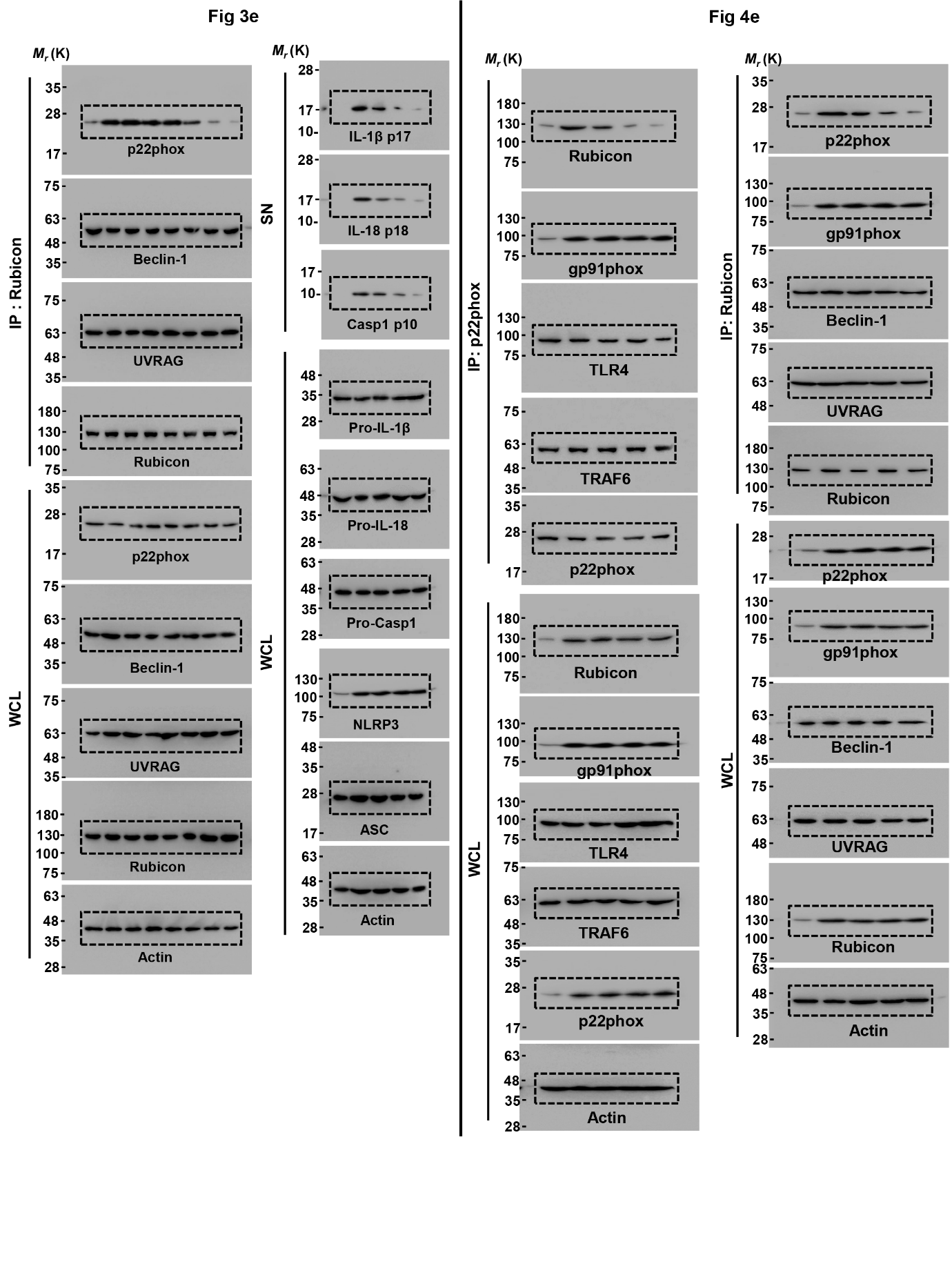
**

**Figure S9. Full-length western blots**

Full-length images of the blots presented in the **Fig. 3e** and **4e**.


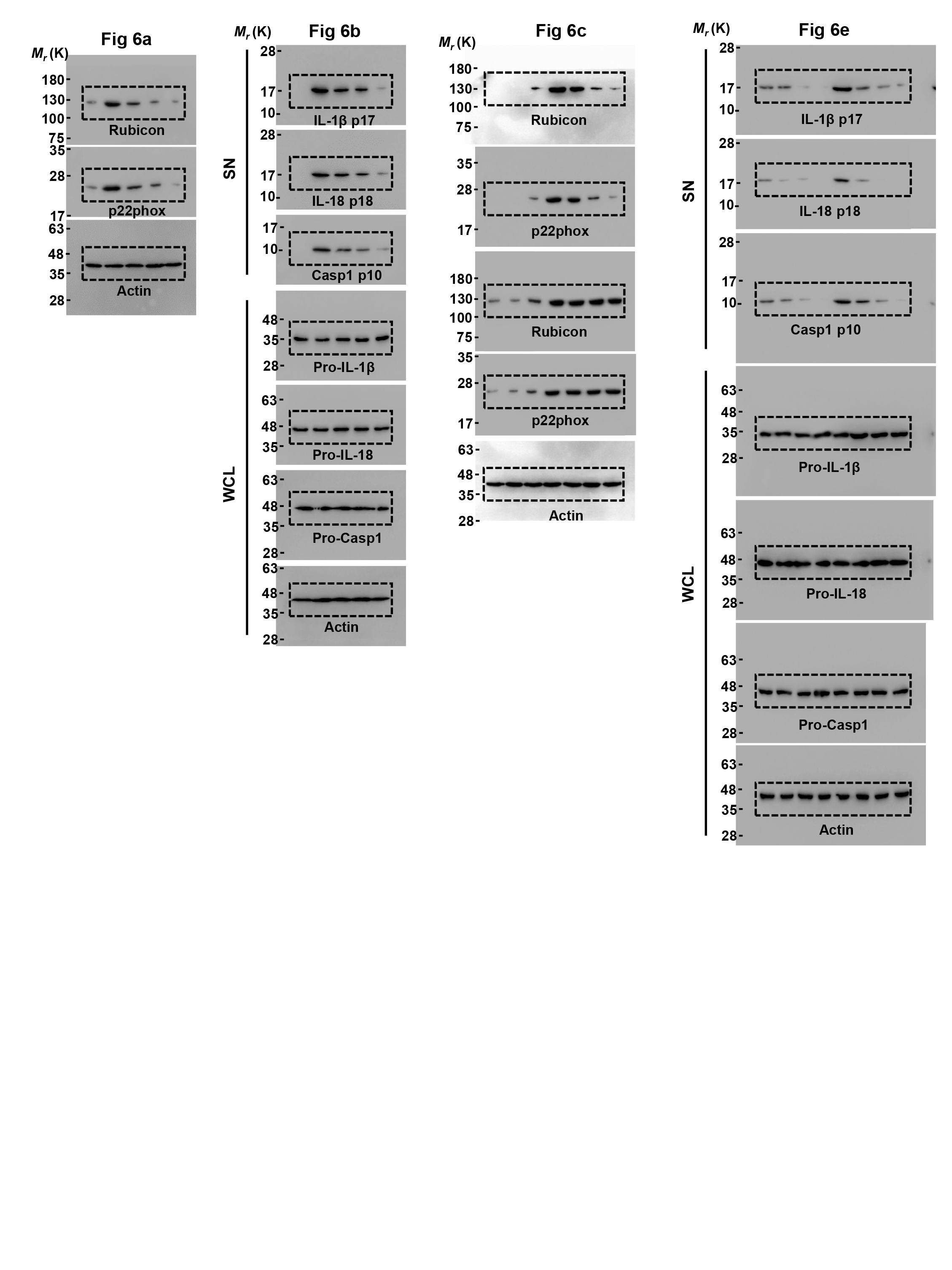


**Figure S10. Full-length western blots**

Full-length images of the blots presented in the **Fig. 6a, 6b, 6c** and **6e**.


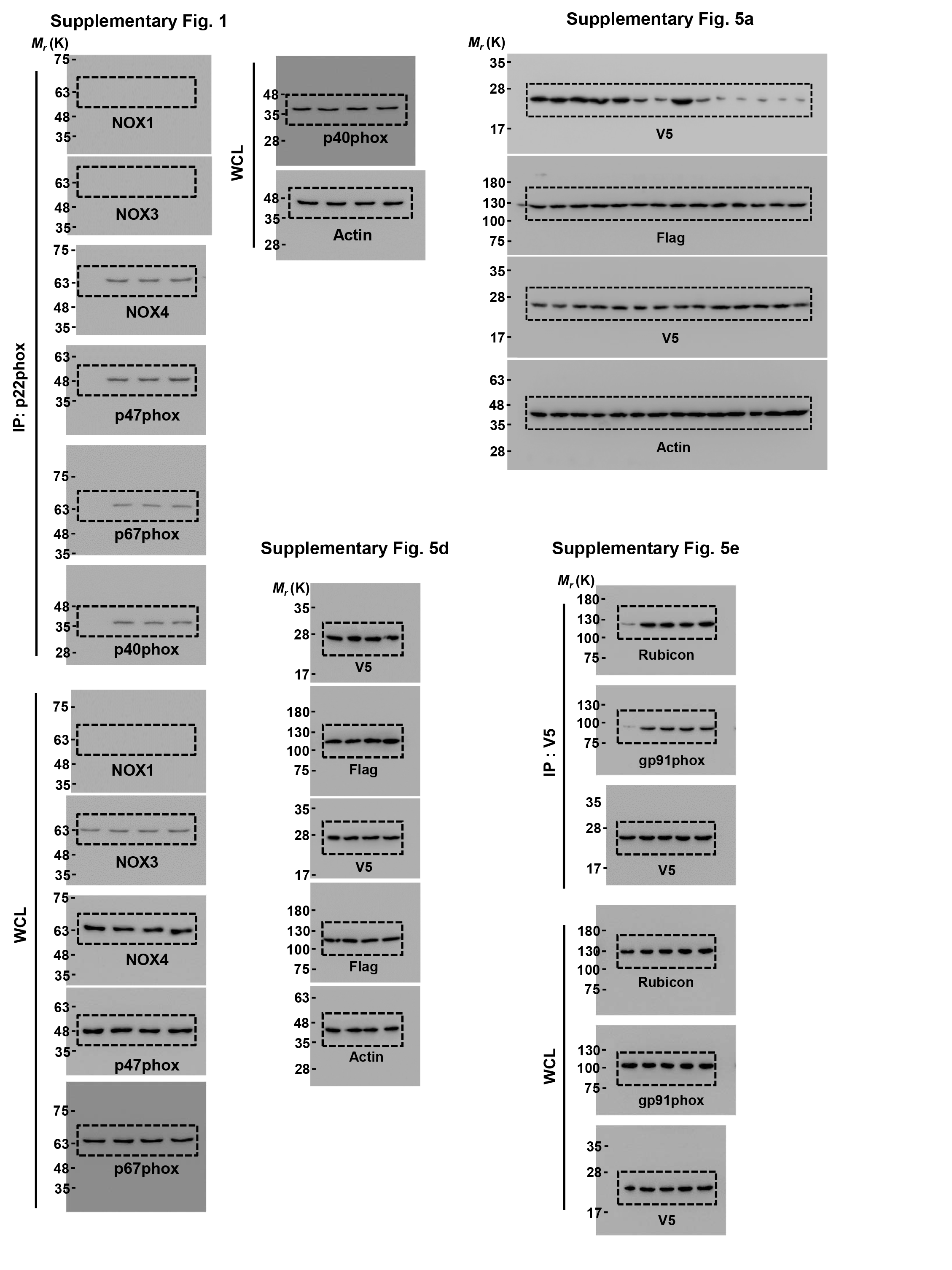


**Figure S11. Full-length western blots**

Full-length images of the blots presented in the **Fig. S1, S5a, S5d** and **S5e**.


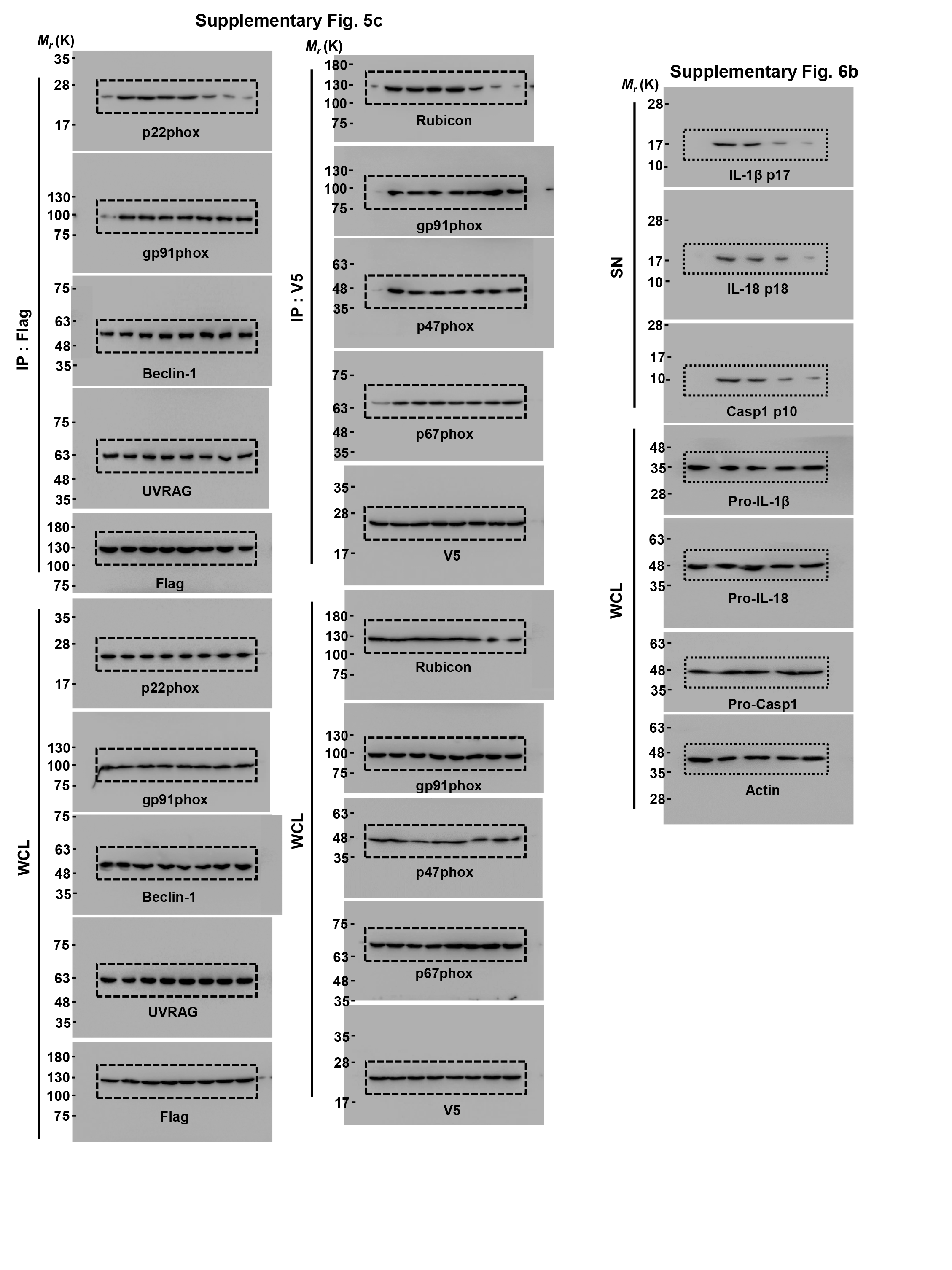


**Figure S12. Full-length western blots**

Full-length images of the blots presented in the **Fig. S5c** and **S6b**.


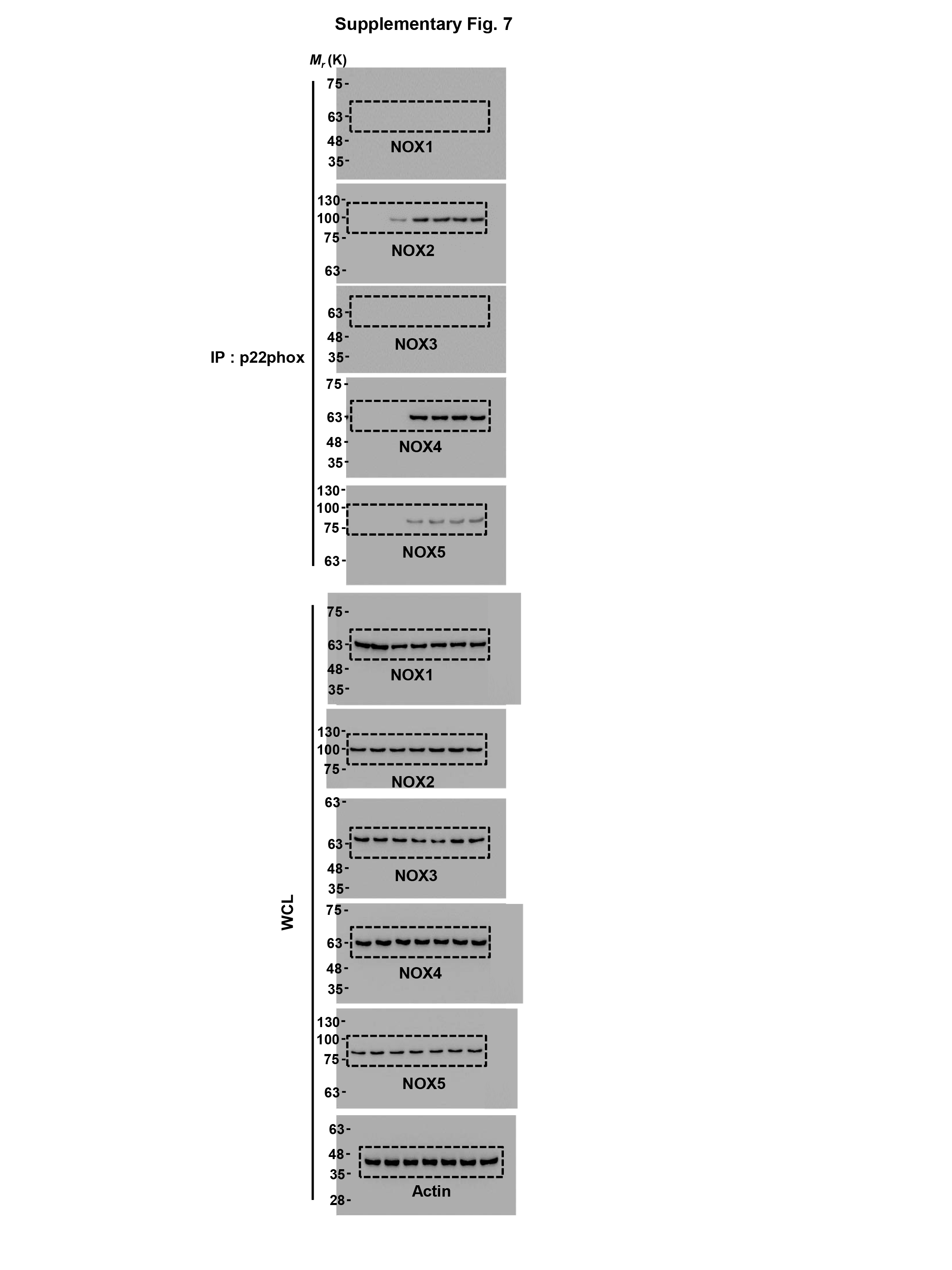


**Figure S13. Full-length western blots**

Full-length images of the blots presented in the **Fig. S7**

**Supplemental Experimental Procedures**

**Synthesis of 2-(tetrahydroindazolyl)phenoxy-*N*-(thiadiazolyl)propanamide (TIPTP)**

All reactions were conducted using oven-dried glassware under an atmosphere of argon (Ar). All commercially available reagents and anhydrous solvents were obtained from Sigma Aldrich, TCI, Alfa, Junsei, Samchun, and DaeJung Chemical and were used without further purification. CH_2_Cl_2_ was dried and distilled following standard protocols. Organic solvents were evaporated with reduced pressure using a rotary evaporator. Reactions were followed by TLC analysis using silica gel 60 F_254_ with fluorescent indicator using UV lamp and KMnO_4_ solution with heat as visualizing agents. Flash chromatography was carried out using Merck silica gel 60 (0.063-0.200 mm) and Kanto silica gel 60N (spherical, neutral). The ^1^H NMR spectra and ^13^C NMR spectra were measured with Bruker AVANCE III HD 400. ^1^H NMR chemical shifts are expressed in parts per million (*δ*) downfield to CHCl_3_ (*δ* = 7.26), ^13^C NMR chemical shifts are expressed in parts per million (*δ*) relative to the central CDCl_3_ resonance (*δ* = 77.0). Coupling constants in ^1^H NMR are in Hz. The following abbreviations were used to designate multiplicities: s= singlet, d= doublet, t= triplet, q= quartet, dd=doublet of doublets, m= multiplet. CDCl_3_ was used as NMR solvent and standard material TMS (tetramethylsilane) wasn’t contained.

**2-(3-Methoxyphenyl)-4,5,6,7-tetrahydro-2*H*-indazole (3)**

To a solution of 4,5,6,7-tetrahydro-2*H*-indazole (1.00 g, 8.18 mmol) in toluene (11 mL) was added 3-iodoanisole (1.20 mL, 9.82 mmol), *trans*-1,2-cyclohexanediamine (0.20 mL. 1.64 mmol), potassium carbonate (2.40 g, 17.2 mmol) and copper (I) iodide (0.080 g, 0.041 mmol). The reaction was heated at 105 °C and stirred for overnight. The resulting mixture was cooled to room temperature, quenched with water (60 mL) and extracted with ethyl acetate (3 x 50 mL). The combined organic layers were washed with brine and concentrated under reduced pressure. The crude residue was purified by silica gel column chromatography (hexane/ethyl acetate = 10:1) to give the title compound (1.10 g, 59%) as solid. ^1^H NMR (400 MHz, CDCl_3_) *δ* 7.59 (s, 1H), 7.27 (t, *J* = 8.1 Hz, 1H), 7.23 (t, *J* = 2.2 Hz, 1H), 7.15 (dd, *J* = 8.0, 1.1 Hz, 1H), 6.75 (dd, *J* = 8.2, 2.4 Hz, 1H), 3.84 (s, 3H), 2.76 (t, *J* = 6.2, 2H), 2.60 (t, *J* = 6.2, 2H), 1.86-1.75 (m, 4H).

**3-(4,5,6,7-Tetrahydro-2*H*-indazol-2-yl)phenol (4)**

To a solution of 2-(3-methoxyphenyl)-4,5,6,7-tetrahydro-2*H*-indazole (1.10 g, 4.82 mmol) in DCM (48 mL) cooled to 0 °C was added BBr_3_ (1.0M in DCM) (9.6 mL, 9.64 mmol). The reaction mixture was stirred at room temperature for 18 h. The resulting mixture was quenched with water (60 mL) and extracted with dichloromethane (3 x 50 mL). The combined organic layers were washed with brine and concentrated under reduced pressure give the title compound (0.89 g, 86%) as solid, which was used in the next step without further purification. ^1^H NMR (400 MHz, CDCl_3_) *δ* 7.56 (s, 1H), 7.27 (br s, 1H), 7.22 (t, *J* = 8.1 Hz, 1H), 7.06-7.04 (m, 1H), 6.73-6.70 (m, 1H), 2.78 (t, *J* = 6.2, 2H), 2.60 (t, *J* = 6.2, 2H), 1.86-1.76 (m, 4H).

**Methyl (*R*)-2-(3-(4,5,6,7-tetrahydro-2*H*-indazol-2-yl)phenoxy)propanoate (5)**

To solution of 3-(4,5,6,7-tetrahydro-2*H*-indazol-2-yl)phenol (0.10 g, 0.47 mmol) in DMF (2.5 mL) was added potassium carbonate (0.13 g, 0.93 mmol) and methyl (*S*)-(-)-2-chloropropionate (0.075 mL, 0.93 mmol). The reaction mixture was heated at 80 °C for 6 h. The resulting mixture was cooled to room temperature, quenched with water (30 mL) and extracted with ethyl acetate (3 x 25 mL). The combined organic layers were purified by column chromatography on silica gel (hexane/ethyl acetate = 3:1) to give the title compound (0.060 g, 39%) as oil. [α]_D_^21^ = +10.9 (*c* = 0.5 in CH_2_Cl_2_). ^1^H NMR (400 MHz, CDCl_3_) *δ* 7.58 (s, 1H), 7.32-7.21 (m, 3H), 6.74-6.71 (m, 1H), 4.86 (q, *J* = 6.8 Hz, 1H), 3.76 (s, 3H), 2.78 (t, *J* = 6.2, 2H), 2.60 (t, *J* = 6.2, 2H), 1.87-1.76 (m, 4H), 1.63 (d, *J* = 6.8, 3H).

**(*R*)-2-(3-(4,5,6,7-tetrahydro-2*H*-indazol-2-yl)phenoxy)propanoic acid (6)**

To a solution of (*R*)-2-(3-(4,5,6,7-tetrahydro-2H-indazol-2-yl)phenoxy)propanoic acid (55 mg, 0.18 mmol) in MeOH (0.9 mL) and H_2_O (0.3 mL) cooled to 0 °C was added lithium hydroxide (6.6 mg, 0.27 mmol). The reaction mixture was stirred at room temperature for 3 h. The solvent was removed under reduced pressure and the residue was diluted with water (5 mL). Next, 1N HCl (1 mL) was added to the resulting solution dropwise. The mixture was extracted with DCM (3 x 15 mL), washed with brine and concentrated under reduced pressure. The crude material was dried *in vacuo* to give the title compound (41 mg, 81%) as solid, which was used in the next step without further purification. [α]_D_^22^ = +16.4 (*c* = 0.5 in CH_2_Cl_2_). ^1^H NMR (400 MHz, MeOD) *δ* 7.98 (s, 1H), 7.39 (t, *J* = 8.5 Hz, 1H), 7.25-7.23 (m, 1H), 6.88 (d, *J* = 8.4, 1H), 4.92 (t, *J* = 6.8, 1H), 2.76 (t, *J* = 6.0, 2H), 2.65 (t, *J* = 6.2, 2H), 1.90-1.81 (m, 4H), 1.61 (d, *J* = 6.8, 3H).

**(*R*)-2-(3-(4,5,6,7-tetrahydro-2*H*-indazol-2-yl)phenoxy)-*N*-(5-(trifluoromethyl)-1,3,4-thiadiazol-2-yl)propanamide (TIPTP, 2)**

To solution of acid (41 mg, 0.14 mmol) in DMF (4 mL) was added *N*,*N*-diisopropylethylamine (0.12 mL, 7.16 mmol), HBTU (110 mg, 0.29 mmol) and 2-amino-5-trifluoromethyl-1,3,4-thiadiazole (24 mg, 0.14 mmol). The reaction mixture was stirred placed at 60 °C for 3 h. The resulting mixture was cooled to room temperature, quenched with water (10 mL) and extracted with ethyl acetate (3 x 15 mL). The combined organic layers were washed with brine and concentrated under reduced pressure. The crude residue was purified by column chromatography on silica gel (hexane/ethyl acetate = 3:1) to give the title compound (24 mg, 39%) as solid. [α]_D_^22^ = -19.6 (*c* = 0.5 in CH_2_Cl_2_). ^1^H NMR (400 MHz, CDCl_3_) δ 7.59 (s, 1H), 7.37~7.24 (m, 3H), 6.79 (dd, *J* = 8.1, 2.4 Hz, 1H), 5.12 (q, *J* = 6.7, 1H), 2.75 (t, *J* = 6.7, 2H), 2.61 (t, *J* = 6.3, 2H), 1.86-1.75 (m, 4H), 1.69 (d, *J* = 6.8, 3H). ^13^C NMR (100 MHz, CDCl_3_) *δ* 207.0, 170.4, 156.8, 151.8, 141.9, 130.7, 123.8, 118.8, 112.5, 106.5, 76.7, 74.3, 30.9, 23.3, 20.6, 18.2. LRMS (ESI) m/z: [M + H]^+^ calcd for C_19_H_18_F_3_N_5_O_2_S 438.1; found 438.1.

**CYP450 assay**

All of CYP inhibition, microsomal stability and plasma stability experiments were performed by New Drug Development Center, the Daegu-Gyeongbuk Medical Innovation Foundation (DGMIF), South Korea. To human liver microsomes (0.25 mg/mL), 0.1 M phosphate buffer (pH 7.4), a cocktail of five probe substrates (Phenacetin 50 μM, Diclofenac 10 μM, S-mephenytoin 100 μM, Dextromethorphan 5 μM, and Midazolam 2.5 μM), and tested compounds were added at concentrations of 0 μM (as a control) and 10 μM. After incubation at 37 °C for 5 min, NADPH generation system solution was also added and incubated at 37 °C for 15 min again. To terminate the reaction, acetonitrile including internal standard (Terfenadine) was added, and the solution was centrifuged for 5 min (14 000 rpm, 4 °C). The supernatant was then injected into the LC-MS/MS system to simultaneously analyze the metabolites of the probe substrates and evaluate the % CYP inhibition of the tested compound.

**Microsomal stability assay**

To human liver microsomes (0.5 mg/mL) were added 0.1 M phosphate buffer (pH 7.4) and tested compounds (1 μM). After incubation at 37 °C for 5 min, NADPH generation system solution was added and incubated at 37 °C for 30 min. To terminate reaction, acetonitrile including internal standard (chloprapamide) was added, and the solution was centrifuged for 5 min (14,000 rpm, 4 °C). The supernatant was then injected into the LC-MS/MS system to analyze the microsomal stability of the tested compound.

**Plasma stability assay**

Human plasma in each culture tube, treated with test compound (10 μM), was incubated at 37 °C for 0, 30 and 120 min. At the determined period of time, internal standard solution of chlopropamide in acetonitrile was added into each culture tube and then tubes were vortexed for 5 min, and then centrifuged for 5 min (14,000 rpm, 4 °C). The supernatant was then injected into LC-MS/MS system to analyze the plasma stability of the tested compound.

**Pharmacokinetic study**

TIPTP at a dose of 0.5 mg/kg and 1 mg/kg was administered intravenously and intraperitoneally to male ICR mice. Blood samples were collected via the carotid artery at 0 (to serve as a control), 0.03, 0.16, 0.25, 0.5, 1, 2, 4, 6, and 24 h after administration of the compound. After centrifugation at 3000 rpm for 10 min, a 13.5 µL aliquot of plasma samples were stored at -20 C until analysis. Pharmacokinetic parameters were determined by a noncompartmental analysis using WinNonlin® (Pharsight Corporation, Mountain View, CA) program. The total area under the plasma concentration-time curve from time zero to the last measured time (AUC_last_) was calculated by the trapezoidal rule-extrapolation method. Standard methods in the literature (M. Gibaldi, D. Perrier, Pharmacokinetics, second ed., Marcel-Dekker, New York, 1982.) were used to calculate the following pharmacokinetic parameters; the time averaged total body clearance (CL), total area under the first moment of plasma concentration-time curve from time zero to time infinity (AUC_0-∞_), terminal half-life, mean residence time (MRT), apparent volume of distribution at steady state (V_ss_). Concentrations of the compound in the above samples were analyzed using LC-MS/MS. To a 13.5 µL aliquot of plasma sample, a 60 µL aliquot of acetonitrile containing 1 µg/mL of internal standard (carbamazepine) was added. After vortex mixing and centrifugation at 6,000 rpm for 15 min, a 100 µL of supernatant was added to 100μL distilled water and injected into LCMS/MS system. The LC-MS/MS system consisted of Agilent 1200 series HPLC system (Agilent, Santa Clara, CA) and API3200® triple-quadrupole mass spectrometer (Applied Biosystems-SCIEX, Concord, Canada). The HPLC mobile phases consisted of 0.1% formic acid in water (A) and acetonitrile (B). Chromatographic separation was achieved on a reversed-phase Atlantis T3 column (100 x 2.1 mm, 3 µm, Waters Corporation, Milford, MA) using gradient elution at a flow rate of 0.25 mL/min. The lower limit of quantitation of the compound in mice plasma was 9.7 ng/mL. The values of coefficients of correlation (R) were more than 0.997.

**Histology and immunohistochemistry**

For immunohistochemistry, tissue sections were fixed in 10% formalin and embedded in paraffin. Paraffin sections (4 μm) were cut and stained with hematoxylin and eosin (H&E). Histopathologic score was established on the basis of the numbers and distribution of inflammatory cells within the tissues, as well as noninflammatory changes such as evidence of bronchiolar epithelial injury and repair ^1,2^. A board-certified pathologist scored each lung section independently without prior knowledge of the treatment groups. A mean score with S.E.M. was calculated for each treatment group. For immunostaining ^2,3^, the 4-μm paraffin sections were deparaffinized and hydrated by serial immersion in 100, 95, and 80% ethanol, distilled water, and PBS. The slides were blocked with 1.5% normal rabbit serum in PBS for 20 min and stained for Rubicon (ab92388; Abcam) or p22phox (CS9; Santa Cruz Biotechnology), followed by peroxidase-conjugated goat αMouse or αRabbit immunoglobulin G (H+L) secondary antibody, and Dako REAL EnVision Detection System, Peroxidase/DAB+ (Dako, Glostrup, Denmark) for immunohistochemistry. For immunofluorescent staining, the secondary antibodies were αMouse-Alexa Fluor®488 and αRabbit-Alexa Fluor®647 (Jackson Immuno Research). Nuclei were stained with DAPI for 1 min. After mounting, fluorescent images were acquired using a confocal laser-scanning microscope (LSM 710; Zeiss, CLSM, Jena, Germany). For colocalization analysis, the co-distribution of the Rubicon and p22phox were quantified and validated statistically by Pearson coefficient, as specified by the ZEN 2009 software (version 5.5 SP1; Zeiss). Imaris 7.1.1 (Bitplane) and Adobe Photoshop 7 (Adobe Systems) were used for image processing and staining scoring.

**Histomorphometric and Histopathologic analysis of arthritis**

Arthritis mice were evaluated for disease progression three times a week as follows ^1,2^. Each limb was scored according to the severity of inflammation, where 0 : no evidence of redness or swelling, 1 : redness and slight swelling confined to the tarsal joints or ankle, 2 : redness and mild swelling extending from the ankle to the tarsal joints, 3 : redness and moderate swelling extending from the ankle to the metatarsal joints, and 4 : severe swelling encompassing the ankle, foot, and digits, or ankylosis of the limb. If the paw is ankylosed, the mouse cannot grip the wire at the top of the cage. Hind paws were fixed for 48 h in 10% buffered formalin and decalcified in 15% EDTA. The paws were then embedded in paraffin, and serial 5-μm sagittal sections of whole hind paws were cut and stained with hematoxylin and eosin. Sections were placed under a microscope and analyzed by 2 observers in a blinded manner for the degree of inflammation and for cartilage and bone destruction according to the method reported previously ^4^, using the following scale: 0 : normal synovium; 1 : synovial membrane hypertrophy and cell infiltrates; 2 : pannus and cartilage erosion; 3 :  major erosion of cartilage and subchondral bone; and 4 : loss of joint integrity and ankylosis based on the extent of infiltration of inflammatory cells into the synovium.

**References**

1 Brand, D. D., Latham, K. A. & Rosloniec, E. F. Collagen-induced arthritis. *Nat Protoc* **2**, 1269-1275, doi:10.1038/nprot.2007.173 (2007).

2 Kim, Y. R. *et al.* Peptide inhibition of p22phox and Rubicon interaction as a therapeutic strategy for septic shock. *Biomaterials* **101**, 47-59, doi:10.1016/j.biomaterials.2016.05.046 (2016).

3 Yang, C. S. *et al.* Autophagy protein Rubicon mediates phagocytic NADPH oxidase activation in response to microbial infection or TLR stimulation. *Cell Host Microbe* **11**, 264-276, doi:10.1016/j.chom.2012.01.018 (2012).

4 Mukai, T. *et al.* Loss of SH3 domain-binding protein 2 function suppresses bone destruction in tumor necrosis factor-driven and collagen-induced arthritis in mice. *Arthritis Rheumatol* **67**, 656-667, doi:10.1002/art.38975 (2015).
